# Supplementary material for: Acute cardiovascular responses of postmenopausal women to resistance training sessions differing in set configuration: A study protocol for a crossover trial
Source: PLoS One. 2024 Oct 14;19(10):e0311524. doi: 10.1371/journal.pone.0311524 (PMC11472946; doi:10.1371/journal.pone.0311524)
Supplement: S1 Protocol — (DOCX) [file pone.0311524.s003.docx]

**Título del proyecto**

Adaptaciones cardiovasculares al ejercicio de fuerza: Efecto de la configuración de la serie en mujeres postmenopáusicas normo e hipertensas.

- **Versión de protocolo:** Versión 1 de fecha 14/07/2022
- **Promotor:** Eliseo Iglesias Soler
- **Investigador principal:** Eliseo Iglesias Soler. Profesor Titular de Universidad. Departamento de Educación Física y Deportiva. Universidade da Coruña.
- **Investigadores/as colaboradores:**
  - **Equipo de Investigación:**
    - Manuel Giráldez García (Departamento de Educación Física y Deportiva. Universidade da Coruña)
    - Xurxo Dopico Calvo (Departamento de Educación Física y Deportiva. Universidade da Coruña)
    - Gonzalo Márquez Sánchez (Departamento de Educación Física y Deportiva. Universidade da Coruña)
    - Alexis Padrón Cabo (Departamento de Educación Física y Deportiva. Universidade da Coruña)
  - **Equipo de Trabajo:**
    - María Rúa Alonso (Departamento de Educación Física y Deportiva. Universidade da Coruña)
    - Jessica Rial Vázquez (Departamento de Educación Física y Deportiva. Universidade da Coruña)
    - Ana Martínez Cagiao (Universidade da Coruña)
    - Lucía Vila Barrios (SERVICIO GALLEGO DE SALUD)
    - Adrián Varela Sanz (Departamento de Educación Física y Deportiva. Universidade da Coruña)
    - Eduardo Carballeira Fernández (Departamento de Educación Física y Deportiva. Universidade da Coruña)
    - Iván Nine Sieira (Departamento de Educación Física y Deportiva. Universidade da Coruña)
    - Enrique Fraga Germade (Departamento de Educación Física y Deportiva. Universidade da Coruña)
    - Pablo Díaz Brage (Universidade da Coruña)

**ÍNDICE**

[Resumen 3](#_Toc178593647)

[Antecedentes y justificación del proyecto 3](#_Toc178593648)

[Hipótesis del estudio 7](#_Toc178593649)

[Objetivos 8](#_Toc178593650)

[Tipo de estudio: 8](#_Toc178593651)

[Material y métodos 8](#_Toc178593652)

[Ámbito de estudio 8](#_Toc178593653)

[Definición de los sujetos a estudio 8](#_Toc178593654)

[Selección y retirada de sujetos 9](#_Toc178593655)

[Captación y reclutamiento de los participantes 9](#_Toc178593656)

[Procedimiento de asignación/aleatorización 9](#_Toc178593657)

[Justificación del tamaño muestral 9](#_Toc178593658)

[Variables principales y secundarias 10](#_Toc178593659)

[Descripción de la intervención 10](#_Toc178593660)

[Cronograma y fecha prevista de finalización. Distribución de las tareas entre los miembros del equipo investigador 12](#_Toc178593661)

[Plan de análisis estadístico 13](#_Toc178593662)

[Aspectos ético-legales 14](#_Toc178593663)

[Cumplimiento de Normas de Buena Práctica Clínica, Declaración de Helsinki, Convenio de Oviedo, así como normativa de protección de datos, manejo de historia clínica y otra normativa de aplicación según el tipo de estudio 14](#_Toc178593664)

[Modelos de consentimiento informado. 14](#_Toc178593665)

[Memoria económica y fuente de financiación 14](#_Toc178593666)

[Bibliografía 15](#_Toc178593667)

# Resumen

La menopausia causa cambios hormonales que afectan a las respuestas fisiológicas agudas y crónicas al ejercicio de mujeres postmenopáusicas. A este respecto, los estudios acerca de las respuestas cardiovasculares al ejercicio de fuerza realizado por mujeres son escasos, siendo especialmente importante identificar los parámetros de la carga que modulan estas adaptaciones. Estudios previos de nuestro grupo de investigación han detectado que la configuración de la serie del ejercicio muscular influye en el estrés cardiovascular implicado, si bien estos resultados fueron obtenidos en sujetos jóvenes y sanos. Por ello, el principal objetivo de este proyecto es ampliar esta línea de investigación y analizar el efecto de la configuración de la serie del ejercicio de fuerza sobre las respuestas cardiovasculares de mujeres postmenopáusicas. Además, dado que estudios previos han mostrado que el nivel de tensión arterial del individuo puede influir en el impacto de programas de entrenamiento de fuerza sobre los cambios cardiovasculares, nos proponemos contrastar dichas modificaciones en mujeres postmenopáusicas normo e hipertensas. El proyecto está orientado a contrastar los efectos de tres sesiones de entrenamiento con el mismo volumen, intensidad, duración y relación trabajo-pausa, pero diferenciados respecto a la configuración de la serie. Para ello, una muestra de 60 mujeres postmenopáusicas (30 normotensas y 30 hipertensas) serán reclutadas en instalaciones deportivas locales para realizar de manera aleatorizada tres sesiones experimentales y una sesión control. Antes y después de cada sesión las respuestas hemodinámicas (tensión arterial sistólica, diastólica y media), de rendimiento cardiaco (frecuencia cardiaca, gasto cardiaco y volumen sistólico entre otros), de modulación autonómica cardiaca y vascular (variabilidad de la frecuencia cardiaca y de la tensión arterial), de efectividad barorrefleja (sensibilidad barorrefleja), de fatiga neuromuscular, de rigidez arterial y metabólicas (lactacidemia, gasto metabólico en reposo mediante calorimetría indirecta) serán valoradas. Este análisis permitirá identificar aquellas configuraciones con mayor y menor estrés cardiovascular. Este proyecto proporcionará información útil para optimizar la prescripción de ejercicio muscular en mujeres postmenopáusicas, mediante la identificación estructuras de esfuerzo que potencialmente permitan, simultáneamente, atenuar el estrés cardiovascular y preservar las adaptaciones fisiológicas.

# Antecedentes y justificación del proyecto

La hipertensión arterial (HTA) o elevación crónica de la presión arterial (PA) es uno de los principales factores de riesgo de enfermedad cardiovascular (ECV), estimándose que es la responsable de 10.8 millones de muertes anuales en el mundo (1). En España, la prevalencia de HTA se sitúa por encima del 40% de la población y aumenta conforme avanza la edad. Así, mientras que en edades medias se han registrado valores del 44%, en mayores de 60 años la prevalencia supera el 75% (2,3). En el caso concreto de población femenina, además de los factores tradicionales de riesgo cardiovascular (4), existen otros específicos (5,6) tras la menopausia: la retirada de los estrógenos se relaciona con cambios en la distribución de la grasa corporal, reducción de la tolerancia a la glucosa, niveles anormales de lípidos, aumentos de la PA, incremento del tono simpático, disfunción endotelial e inflamación vascular… (7). Por esto que, aunque la prevalencia de la HTA entre las mujeres premenopáusicas tiende a ser menor que la de los hombres de edad similar, se vuelve mayor en las mujeres después de la menopausia (5), alcanzando los valores previamente mencionados (3). Además, los cambios hormonales en las mujeres postmenopáusicas aumentan la probabilidad de sufrir osteoporosis, cáncer, problemas a nivel del aparato urinario y del suelo pélvico y demencia, entre otros. Los efectos simultáneos de los cambios hormonales y del incremento de la edad tras la menopausia (envejecimiento) y la infrarrepresentación general de la mujer en la investigación (8,9,10), requieren prestar mayor atención al estudio de este grupo de población.

El tratamiento no farmacológico de la HTA a través de la modificación de hábitos de vida es la primera opción terapéutica, y la reducción del sedentarismo junto a la implantación de programas de ejercicio físico se han mostrado altamente efectivos (11–14). Por su relevancia en la mejora del sistema cardiovascular y la clara asociación entre una buena aptitud aeróbica y la reducción del riego relativo de mortalidad por ECV (15), la atención se ha centrado mayoritariamente en la promoción y la investigación del trabajo aeróbico o cardiorrespiratorio. Sin embargo, la evidencia emergente sobre los beneficios que se derivan de las adaptaciones al trabajo de fuerza, a nivel estructural (prevención de sarcopenia u osteoporosis), funcional (mejora de la movilidad, incremento de la actividad física diaria, reducción del riesgo de caídas), de función cognitiva y salud mental, metabólico (disminución de hemoglobina glicosilada, incremento del metabolismo basal, aumento de masa libre de grasa, mejora del perfiles lipídico) o cardiovascular (reducción de la presión arterial, mejora del control autonómico), así como la reducción de la mortalidad y morbilidad (16–18), ha determinado que el desarrollo del componente muscular a través de ejercicios orientados al desarrollo de la fuerza, haya ido adquiriendo relevancia en los programas de ejercicio físico para la salud. De hecho, muy recientemente, la Sociedad Europea de Cardiología ha incluido el entrenamiento de fuerza entre sus recomendaciones de actividad física y ejercicio (19).

El efecto sobre la disminución de la PA de los programas orientadas al desarrollo de la fuerza muscular mediante ejercicios dinámicos son significativos (20–26), si bien de menor magnitud que los obtenidos mediante otros modelos de intervención como el ejercicio aeróbico o el entrenamiento isométrico de baja intensidad (21). Sucesivos meta-análisis han cuantificado en alrededor de 3 mmHg la reducción media tanto de PA sistólica (PAS) como diastólica (PAD) mediante programas de ejercicios dinámicos con sobrecargas, valores que, aunque modestos, pueden conllevar desde el punto de vista epidemiológico un descenso de entre el 5 y el 9% en la morbilidad cardiaca, entre un 8 y un 14% en el riesgo de enfermedad vascular cerebral o en un 4% el riesgo de mortalidad (12,27–29).

Además, se ha evidenciado que la mejora de la fuerza a través del ejercicio con sobrecargas atenúa la respuesta hemodinámica al manejo de magnitudes absolutas de carga (30), por lo que se sugiere que puede tener un efecto en la mejora del rendimiento muscular y una consecuente reducción en las demandas cardiacas de las actividades cotidianas (18). El entrenamiento dinámico de fuerza también promueve adaptaciones vasculares al mejorar la conductancia vascular y la función endotelial, lo que podría tener un papel en el efecto reductor de la PA de este tipo de entrenamiento (31). A pesar de estas evidencias, los diferentes meta-análisis que han abordado el impacto del trabajo de fuerza sobre los niveles basales de PA coinciden en señalar la necesidad de profundizar en el conocimiento de este fenómeno dado el número limitado de estudios existentes en la actualidad, particularmente en lo referido a aquellos llevados a cabo en población hipertensa (12,27,29,31,32). Valga de ejemplo que en el meta-análisis de Cornelissen et al. (21) fue posible incluir tan solo 29 grupos correspondientes a programas de ejercicio de musculación dinámico, frente a los 105 grupos considerados para el análisis de los efectos de programas de ejercicio aeróbico. Por otro lado, el efecto del entrenamiento de fuerza en la mujer postmenopáusica ha mostrado efectos positivos para la reducción de la PA (33). Sin embargo, tal y como se puede observar en la reciente revisión de Loaiza-Betancur et al. (33), la presencia de estudios específicos sobre las adaptaciones cardiovasculares al ejercicio de fuerza en mujeres postmenopáusicas es escasa y se reduce todavía más si se trata de mujeres con patología hipertensiva; la mayor parte de los estudios incluyen a mujeres sedentarias y hasta la fecha solo se conoce un estudio que investigue los efectos del ejercicio físico en mujeres postmenopáusicas físicamente activas. Esto refleja cierto grado de desconocimiento sobre las adaptaciones al entrenamiento de fuerza en este grupo poblacional especifico que, a priori, también podría beneficiarse de él.

Una evaluación global del efecto cardiovascular de los programas orientados al desarrollo de la fuerza requiere que se consideren, junto a las adaptaciones crónicas, las respuestas hemodinámicas agudas. En este sentido, el fenómeno de la Hipotensión Postejercicio (HPE), es decir, el descenso de la PA a niveles inferiores a los medidos de manera previa al comienzo de la sesión, ha sido observado tanto tras actividades de carácter aeróbico como en rutinas para el desarrollo de la fuerza, particularmente en población hipertensa (34–38), y conlleva que el ejercicio físico sea contemplado como una herramienta útil para el control no farmacológico de la HTA (12–14).

Como será señalado más adelante, nuestro grupo ha desarrollado una línea de investigación en la que se exploran respuestas y adaptaciones cardiovasculares al ejercicio de fuerza, analizando, entre otras cuestiones, los factores que influyen en la TA en población joven, sana activa y fundamentalmente masculina (39–46). Por lo tanto, surge la necesidad de extender nuestros hallazgos a otros perfiles poblacionales, potencialmente diferentes en cuanto a sus respuestas y adaptaciones fisiológicas como es la de mujeres activas postmenopáusicas.

A pesar de los beneficios agudos y crónicos que se derivan de los programas orientados al desarrollo de la fuerza muscular, existe cierta precaución a la hora de implementarlos en algunas poblaciones, porque el trabajo con sobrecargas conlleva una pronunciada respuesta hemodinámica durante su realización (47,48), lo que podría suponer un factor de riesgo que debe ser considerado en pacientes con algún tipo de patología cardíaca (49,50). En la práctica habitual, el entrenamiento de fuerza muscular consta de una o varias series de repeticiones de uno o varios ejercicios. Durante la ejecución de repeticiones sucesivas en una misma serie se produce una pérdida progresiva de rendimiento mecánico (potencia, velocidad, etc.) y, paralelamente, un incremento continuado en la PA, tanto de la PAS como de la PAD, que alcanza su valor pico en el instante en que tiene lugar el fallo muscular (47,48,51,52). De forma simultánea también aumenta la frecuencia cardiaca (FC) y, consecuentemente, se eleva el Doble Producto (DP = FC x PAS) (47,48). A pesar de que el incremento de la PAS en una contracción aislada es dependiente de la intensidad de la misma (53), se ha evidenciado que la duración de la serie es el principal factor que influye en esa elevación (54–59). Así, las series de baja intensidad con un elevado número de repeticiones producen respuestas cardiovasculares más elevadas si se comparan con las series de mayor intensidad, pero con un menor número de repeticiones (54–59). A pesar de ello, en el ámbito de la prescripción de ejercicio con sobrecargas para personas con enfermedades cardiovasculares, sigue predominando la recomendación de realizar ejercicios con pesos ligeros y numerosas repeticiones (18,60). Dado que el nivel de desarrollo de la fuerza depende de manera importante del estímulo mecánico generado durante los ejercicios (fuerza, potencia, tensión muscular), y que la prolongación temporal de las series de trabajo puede conllevar una respuesta hemodinámica más acentuada, es posible deducir que las pautas recomendadas actualmente para sujetos con patología cardiovascular pueden tener efectos limitados sobre la mejora de la fuerza y, al mismo tiempo, suponer un mayor estrés cardiovascular durante la práctica del ejercicio de fuerza, con todos los riesgos que esto comporta.

Surge, por lo tanto, la necesidad de diseñar vías de intervención que conjuguen el incremento en la calidad del estímulo mecánico con la reducción de las respuestas cardiovasculares durante el ejercicio. En este sentido, frente al modelo tradicional de ejercitación, que consiste en la realización continuada de las repeticiones del ejercicio hasta niveles próximos al fallo muscular, aparece un diseño alternativo de la serie de trabajo, basado en modificar los períodos de trabajo y pausa, fraccionando la serie en pequeños grupos de repeticiones. Este tipo de entrenamiento ha recibido diferentes denominaciones: Cluster Training, Inter-repetition rest training o Intra-Set Rest loading (61). A este respecto, diferentes trabajos de nuestro grupo han evidenciado cómo configuraciones de la serie más cortas, tipo clúster, conllevan una reducción de la percepción de esfuerzo (62,63) y un mejor rendimiento mecánico durante la sesión en términos de velocidad y potencia (64–69). La configuración de la serie tipo clúster implicaría un menor tiempo de compresión mecánica de los vasos musculares o del tiempo e intensidad de realización de la maniobra de Valsalva, factores responsables de la respuesta hemodinámica durante el ejercicio de fuerza (48). Estudios previos han demostrado que la inclusión de pequeñas pausas de relajación entre repeticiones es una estrategia eficaz para reducir los incrementos de la PA durante el ejercicio con sobrecargas (70–72). No obstante, estos trabajos no equipararon la relación entre trabajo y pausa, de tal manera que las sesiones con descansos entre repeticiones acumularon mayor tiempo de recuperación y por lo tanto aumentaron la duración de las sesiones. Estas desigualdades entre las configuraciones podrían haber tenido alguna incidencia en los resultados. Se hace necesario por ello, valorar el efecto de la configuración de la serie (tipo clúster vs. entrenamiento tradicional) aislando este factor de aquellos otros que pudiesen incidir sobre los resultados. Esto es, valorar el efecto de la configuración de la serie requiere que el resto de los factores de la carga (volumen, intensidad, tiempo total de recuperación) permanezcan estables entre condiciones. Nuestro grupo ha trabajado en los últimos años sobre esta línea de investigación, observando que, en comparación con una configuración tradicional, las configuraciones de la serie relativamente cortas (es decir, una estructura de la serie donde se realicen en torno al 30-40% de las repeticiones máximas), son las que atenúan la respuesta de la PA (44). No obstante, estas valoraciones se obtuvieron en una muestra de población joven, sana y físicamente activa (estudiantes de Ciencias de la Actividad Física y del Deporte) y predominantemente de sexo masculino. En un trabajo reciente con pacientes coronarios, en el que participó el IP de este proyecto (73), también se ha verificado esta atenuación del estrés cardiovascular con configuraciones cortas de la serie, aunque en este caso la muestra era exclusivamente masculina. Sería por ello oportuno implementar este tipo de análisis en población femenina y, de manera particular, en mujeres postmenopáusicas y diagnosticadas con hipertensión. La evidencia apunta a que este grupo poblacional se podría beneficiar en gran medida de estructuras de la serie más cortas, ya que aportarían los beneficios de entrenamiento de fuerza con un menor riesgo cardiovascular. Además, considerando las respuestas adversas que se pueden observar durante la menopausia (fatiga, cansancio, etc.), estos protocolos con series cortas generan menores percepciones del esfuerzo durante el ejercicio y podrían favorecer la adherencia a los programas de entrenamiento.

Las respuestas hemodinámicas al ejercicio están vinculadas a las variaciones del control cardiaco y vascular realizadas, en gran medida, por el Sistema Nervioso Autónomo (SNA). Actualmente, el control autonómico cardiaco puede evaluarse de manera no invasiva a través del análisis de factores como la variabilidad de la frecuencia cardiaca (VFC) o la complejidad de la frecuencia cardiaca (CFC), mientras que el tono vascular simpático puede ser estimado mediante análisis de la variabilidad de la presión arterial (VPA) (74,75). De forma complementaria, el análisis conjunto latido a latido de la PAS y de la FC permiten la evaluación de la sensibilidad barorrefleja (SBR), entendida como la eficacia del barorreflejo para cambiar el ritmo cardiaco en respuesta a los cambios en la PAS (76). Pocos estudios han analizado el impacto del trabajo de fuerza en el control autonómico cardiaco y la SBR. Trabajos previos han mostrado una pérdida transitoria de control autonómico cardiaco (77,78) y una disminución de la SBR de manera inmediatamente posterior a la finalización de una sesión de ejercicios con sobrecargas (79–83). Estas reducciones de ambas variables provocan una situación de vulnerabilidad, porque el riesgo de sufrir ciertos eventos cardiovasculares está asociado a bajos niveles de VFC y SBR, especialmente en población con riesgo cardiovascular (84–87).

Nuestro grupo de investigación ha sido pionero en el análisis del efecto de la configuración de la serie sobre los cambios agudos en la VFC (39–45). Los resultados de estos trabajos muestran una atenuación de la pérdida del control autonómico cuando se emplean configuraciones de serie cortas tipo clúster, en comparación con estructuras tradicionales próximas al fallo muscular. Esto sugiere el uso de la configuración de la serie como elemento regulador de las modificaciones en la modulación autonómica cardiaca inducidas por el ejercicio de fuerza. De nuevo, todos estos trabajos fueron desarrollados con muestras jóvenes, sanas y de sexo masculino, por lo que resultaría oportuno confirmar sus hallazgos en otros perfiles poblacionales como el que es objetivo de este proyecto. A su vez, se ha sugerido que la disminución del SBR posterior al trabajo de fuerza puede venir condicionada por una reprogramación del reflejo derivado de los picos de TA alcanzados durante el ejercicio (81). Por ello es posible establecer la hipótesis de que aquellos diseños de entrenamiento que generen una menor respuesta hemodinámica, también generarán un menor deterioro en la SBR y, en consecuencia, serán protocolos más seguros, con menor riesgo cardiovascular. Esta cuestión también ha sido abordada en los trabajos mencionados con anterioridad, en los que se observó una menor disminución de la SBR tras la ejecución de rutinas de ejercicio con configuraciones cortas tipo clúster en comparación con estructuras más convencionales. Por lo tanto, nuestros trabajos parecen indicar también un menor deterioro de la SBR postejercicio en población joven y sana tras el trabajo con estructuras tipo clúster. Todo esto permite concluir que las configuraciones de la serie cluster o cortas constituyen configuraciones de esfuerzo que permiten aunar la mejora de la estimulación mecánica del músculo, la disminución del estrés hemodinámico durante la sesión, una atenuación de la pérdida aguda de modulación parasimpática cardiaca y barorrefleja, convirtiéndolas en estrategias de entrenamiento con un balance beneficio-riesgo más positivo. Sin embargo, tal y como se ha mencionado previamente, no es posible extrapolar estas conclusiones a otros grupos poblacionales más allá de la población joven, sana y físicamente activa. Son los colectivos con más edad y con patología cardiovascular los que podrían beneficiarse en mayor medida de todas las ventajas de este tipo de estructuras. Un claro ejemplo, teniendo en cuenta lo previamente señalado y la ausencia de evidencia suficiente, serían las mujeres postmenopáusicas con y sin hipertensión arterial, dado que ambos factores (menopausia e hipertensión) configuran un perfil fisiológico diferenciado en el que coexisten los efectos negativos de los cambios hormonales y el transcurso de la edad. Esto señala a este grupo de población como candidato para beneficiarse de investigaciones que permitan identificar qué modelos de entrenamiento de la fuerza podría aportar el mayor beneficio para su salud y calidad de vida con el menor riesgo cardiovascular asociado, de modo que la práctica de ejercicio físico sea una tratamiento no farmacológico seguro para el manejo de la HTA y otras consecuencias específicas que se asocian con la menopausia (osteoporosis, sarcopenia…).

# Hipótesis del estudio

1. Basándonos en los hallazgos previos de nuestro grupo, una atenuación de las respuestas hemodinámicas y del deterioro del control autonómico y barorreflejo cardiaco tras la realización de ejercicio muscular con configuraciones de serie cortas comparada a las provocadas por configuraciones más convencionales.
2. Basándonos en estudios previos que señalan una mayor reducción de la PA en personas hipertensas, y tras configuraciones largas próximas al fallo muscular (43), esperamos un efecto hipotensivo del ejercicio de fuerza especialmente tras configuraciones largas más fatigantes y particularmente en la muestra de mujeres hipertensas.

# Objetivos

*OBJETIVOS GENERALES*

- Evaluar el efecto de la configuración de la serie del ejercicio de fuerza sobre las respuestas hemodinámicas y metabólicas agudas, y sobre los mecanismos reguladores (control autonómico cardiaco, tono simpático vasomotor) en mujeres activas postmenopáusicas normo e hipertensas.
- Contrastar las respuestas hemodinámicas agudas entre mujeres postmenopáusicas normo e hipertensas.

*OBJETIVOS ESPECÍFICOS*

- Evaluar el efecto de la configuración de la serie sobre los cambios agudos en la hemodinámica, en el gasto energético en reposo, en el control autonómico cardiaco y vascular de mujeres postmenopáusica normo e hipertensas.
- Determinar el modelo de configuración de la serie que genera una menor alteración hemodinámica y del control neural cardiaco de mujeres postmenopáusicas normo e hipertensas.
- Identificar la configuración de la serie con menor impacto en la disminución de la sensibilidad barorrefleja (SBR) post-ejercicio de mujeres postmenopáusicas normo e hipertensas.
- Evaluar la asociación entre los cambios hemodinámicos durante el ejercicio y las modificaciones agudas de control autonómico y barorreflejo cardiaco de mujeres postmenopáusicas normo e hipertensas.
- Identificar la configuración del ejercicio de fuerza que optimice el fenómeno de la hipotensión post-ejercicio en mujeres postmenopáusicas normo e hipertensas.
- Contrastar el impacto cardiovascular de las diferentes configuraciones de la serie en el ejercicio de fuerza entre mujeres postmenopáusicas normo e hipertensas.

# Tipo de estudio:

- Diseño de medidas repetidas cruzado (*Crossover design*) ya que cada sujeto de la muestra pasará por todas las condiciones experimentales.

# Material y métodos

## Ámbito de estudio

El presente proyecto corresponde al ámbito de las Ciencias de la Actividad Física y del Deporte.

## Definición de los sujetos a estudio

La muestra estará constituida por mujeres postmenopáusicas usuarias de dos instalaciones situadas en la zona de influencia de la ciudad de A Coruña: Termaria Casa del Agua (<https://termaria.es/>) y DOS Acea da Ma (<https://www.dosdeporte.es/inicio-acea-de-ama/>). Tal y como se refleja en el CVA del IP, ambas instalaciones han albergado algunos de los proyectos de transferencia del grupo de investigación y cuentan con convenios activos con la Universidade da Coruña. Asimismo, el grupo de investigación ha contactado con la empresa encargada de la gestión de centros deportivos en el área de Oleiros, próxima a la Facultad en la que se desarrollará el presente proyecto (Aqualia: <http://www.centrosdeportivosoleiros.es/oleiros/>) para localizar muestra entre sus usuarias.

## Selección y retirada de sujetos

Todas las integrantes de la muestra se someterán a una consulta médica previa para evaluar las posibles contraindicaciones absolutas y relativas para la práctica de ejercicio y los criterios de inclusión y exclusión. Esta selección será supervisada por el Dr. Manuel Giráldez García, miembro del equipo de investigación y médico especialista en medicina de la Educación Física y del Deporte. Los criterios de inclusión serán: para todas las mujeres, haber transcurrido 5 años desde la menopausia (se comienzan a contar tras 12 meses consecutivos sin menstruación), tener una edad entre 55 y 64 años, ser físicamente activa (150-300 min/semana de intensidad ligera o 75-150 min/semana de intensidad moderada-vigorosa), no tener más de 3 factores de riesgo cardiovascular, estar asintomática y sin enfermedad cardiovascular, metabólica o renal conocida y disponer de certificado COVID de la UE vigente; para las mujeres con HTA, además de los anteriores, estar diagnosticadas de HTA grado I bien controlada con no más de un fármaco y con riesgo cardiovascular bajo o moderado (*Hypertension*. 2020;75:1334-1357. DOI: 10.1161/HYPERTENSIONAHA.120.15026.). Los criterios de exclusión serán: padecer cualquier otro grado de HTA, necesitar más de un fármaco para controlar la HTA o usar cualquier otro que interfiera con las respuestas cardiovasculares al ejercicio, estar o haber estado sometida a terapia hormonal sustitutiva y presentar respuesta hipertensiva al ejercicio. Cada participante deberá firmar el correspondiente documento de consentimiento informado (DCI) en el que se detallarán todas las características del estudio y que seguirá el modelo sugerido por la Red Gallega de Comités de Ética de la Investigación (<https://acis.sergas.es/cartafol/05-Modelos-de-documentos>). La presente solicitud, va acompañada del citado modelo ajustado a cada uno de los protocolos propuestos. Tal y como se recoge en el mencionado documento, todas las participantes podrán causar baja en el experimento, si así lo desean.

## Captación y reclutamiento de los participantes

La muestra será localizada por parte de los componentes del equipo de investigación, a través de la colocación de cartelería, hoja de inscripción y código QR en la recepción y tablones de las instalaciones que participan en el proyecto. Además, se creará un perfil del proyecto en las principales redes sociales (Twitter/X, Instagram, Facebook) con los criterios de inclusión y se anunciará a través de la página web del grupo de investigación.

## Procedimiento de asignación/aleatorización

Se trata de un diseño de medidas repetidas cruzado, en el que el orden de realización de las diferentes condiciones experimentales será aleatorizado.

## Justificación del tamaño muestral

Se ha realizado un cálculo del tamaño muestral para una potencia estadística del 80%, a un nivel de significación de 0.05, para detectar un tamaño del efecto pequeño (f=0.12) para la interacción entre un factor de medidas repetidas (3 configuraciones y sesión control) y un factor inter-sujeto (grupo normo e hipertenso) y asumiendo una correlación entre medidas repetidas de 0.75. El resultado del cálculo, utilizando el programa G*Power v3.1.9.7, es de una muestra total de 50 mujeres (25 cada grupo). Con este resultado, nos proponemos una muestra total de 60 mujeres postmenopáusicas: 30 de ellas normotensas y 30 hipertensas.

## Variables principales y secundarias

| *Variables principales:* | *Variables secundarias:* |
| --- | --- |
| - Tensión arterias sistólica, diastólica y media | - Edad |
| - Sensibilidad barorrefleja | - Estatura |
| - Variabilidad de la frecuencia cardiaca | - Masa corporal |
| - Variabilidad de la tensión arterial | - Composición corporal |
| - Rigidez arterial | - Densidad mineral ósea |
| - Consumo de oxígeno en reposo | - Años de menopausia |
| - Gasto cardiaco | - Tratamiento en caso de hipertensión |
| - Volumen sistólico | - Carga de 12 repeticiones máximas |
| - Resistencia periférica total en reposo | - Velocidad de ejecución de ejercicios |
| - Lactacidemia | - Resultados de electrocardiograma en reposo |
| - Percepción de esfuerzo | - Consumo máximo de oxígeno |

## Descripción de la intervención

*DISEÑO*: Atendiendo a los objetivos del trabajo, cada componente de la muestra llevará a cabo, en una secuencia aleatorizada, tres sesiones de ejercicio y una sesión de control. La sesión de ejercicio consistirá en completar el mismo volumen de trabajo (repeticiones x kilogramos) en los ejercicios de prensa de piernas, press banca, curl de pierna y jalón al pecho, pero respondiendo a estructuras diferentes respecto a la configuración de las series. Así, con la carga de 12 repeticiones máximas (12RM) se completará un volumen en cada ejercicio equivalente a 3 series de 12 repeticiones en cada uno de los ejercicios, variándose la estructura del esfuerzo en cada una de las sesiones: sesión con 9 series de 4 repeticiones (4S), es decir, al 33% de intensidad de esfuerzo (4 sobre 12 repeticiones posible); sesión de 6 series de 6 repeticiones (6S), y por lo tanto 50% de intensidad de esfuerzo (6 sobre 12 posibles) y finalmente sesión de 4 series de 9 repeticiones (9S) correspondiente al 75% de intensidad de esfuerzo. Para que la relación trabajo/pausa entre sesiones sea equivalente, la recuperación entre series será de 45, 72 y 120 segundos para 4S, 6S y 9S respectivamente. La pausa entre ejercicios será de 4 minutos para todas las sesiones. Este procedimiento asegura la igualdad entre sesiones en cuanto al volumen, intensidad de carga, tiempo de recuperación y duración de la sesión, siendo la variable independiente la configuración de la serie, es decir, la intensidad de esfuerzo requerida. Asimismo, se llevará a cabo una sesión de control (CON), sin ejercicio, al objeto de contar con una referencia "baseline" del comportamiento hemodinámico y metabólico de la muestra a lo largo del tiempo. La estructura para el registro será la misma en 4S, 6S y 9S: **i)** registro inicial durante 15 minutos en reposo de variables hemodinámicas, metabólicas (consumo de oxígeno en reposo), indicadores de modulación autonómica cardiaca, rendimiento cardiaco y medición de rigidez arterial; **ii)** fase de calentamiento de 10´; **iii)** valoración neuromuscular para control de fatiga; **iv)** registro basal de lactato; **v)** realización de la sesión con una duración aproximada de 45´; **vi)** medición de lactato post-ejercicio; **vii)** medición neuromuscular tras ejercicio; **viii)** medición de rigidez arterial; **ix)** repetición del registro hemodinámico, metabólico y de modulación autonómica cardiaca en reposo durante 60´ al objeto de valorar el efecto hipotensivo del ejercicio y **x)** medición final de rigidez arterial final. Por su parte, CON consistirá en la repetición de la estructura anterior, sustituyendo la rutina de ejercicio por 45´ de reposo. Toda esta fase del proyecto se realizará en las instalaciones de la Facultad de Ciencias del Deporte y la Educación Física de la Universidade da Coruña.

*PROCEDIMIENTOS:* Cada sujeto llevará a cabo un total de 8 sesiones de valoración: **Sesión 1.** Realización de consulta médica (Manuel A. Giráldez García) para evaluar el estado de salud, los posibles factores de riesgo cardiovascular, las contraindicaciones (absolutas y relativas) y los criterios de inclusión y exclusión; dicha consulta consistirá en anamnesis (incluyendo informes médicos previos que se aporten), exploración física exhaustiva y pruebas complementarias (previo consentimiento informado): ECG de reposo, espirometría de reposo y ergoespirometría con monitorización de ECG, gases y TA. **Sesión 2**. Se realizarán las mediciones antropométricas (peso, estatura, índice de masa corporal, composición corporal por bioimpedancia, registro de pliegues y diámetros), densidad mineral ósea mediante un osteodensitómetro por ultrasonidos (Sonost 3000, Osteosys Corp., Korea) y la familiarización de cada sujeto con la realización de los ejercicios. Dichos ejercicios se han seleccionado de acuerdo con las recomendaciones internacionales respecto al uso de ejercicios poliarticulares con implicación de grandes grupos musculares. **Sesiones 3 y 4.** Estarán dedicadas a la determinación, para cada ejercicio, de la carga con la que el sujeto es capaz de realizar un máximo de 12 repeticiones (12RM). Durante el procedimiento se registrará la velocidad de ejecución mediante el dispositivo T-Force, al objeto de emplear la pérdida de velocidad propulsiva como orientación a la hora de ajustar las cargas. El procedimiento se llevará a cabo en dos ocasiones de cara a determinar el grado de replicabilidad de los resultados. Una diferencia de más del 10% entre las cargas 12RM definidas en cada sesión, implicará llevar a cabo una tercera valoración. Las sesiones estarán separadas por al menos 72 horas de recuperación. **Sesiones 5-8.** Corresponden a la realización, de forma aleatorizada, de las sesiones 4S, 6S, 9S y CON. Los pasos a seguir en cada sesión serán los siguientes: Una vez que el sujeto acude al laboratorio, es preparado para el registro mediante el dispositivo Task Force Monitor (CNSystems, Graz, Austria) de ECG, presión arterial latido a latido y cardiografía de impedancia. Asimismo, se dispondrá la medición indirecta del gasto metabólico en reposo (consumo de O2) mediante analizador MetaMax 3B-R2 (Cortex, Leipzig, Germany). Una vez realizadas las calibraciones, el registro comienza, manteniéndose el sujeto durante 15´ en tendido supino en una camilla, en silencio. Finalizado este periodo se procederá a valorar el stiffness o rigidez arterial mediante la técnica de medición de la velocidad de la onda de pulso (*finger-toe pulse wave velocity*). Posteriormente, se inicia la fase de calentamiento, consistente en pedalear durante 5´ en un cicloergómetro a una intensidad moderada (50-70% de su frecuencia cardiaca máxima estimada), en 2´ de movilidad articular, y en la realización de 10 contracciones de cada ejercicio con el 70% de la carga que será manejada en la sesión a una velocidad de ejecución moderada. Tras 2´ de recuperación, se hace un registro basal de lactacidemia y se registrará, para cada ejercicio, y mediante transductor de velocidad T-Force, la velocidad de desplazamiento de una carga equivalente al 70% de 12RM. Esta valoración se empleará como indicador de fatiga en cada grupo muscular. A continuación comenzará la sesión de ejercicio, siguiendo las pautas anteriormente descritas. Durante esta fase, además de la medición de parámetros cardiovasculares, será registrado el rendimiento mecánico en cada repetición mediante el dispositivo T-Force así como la percepción de esfuerzo al final de cada serie mediante escala OMNI-RES. Un minuto tras la finalización de la última repetición de la sesión, se realizará una nueva medición de lactacidemia, se indicará al sujeto que se sitúe de nuevo en la camilla, se procederá a la medición de la rigidez arterial, se realizará la preparación de registro cardiovascular mediante el dispositivo Task-Force Monitor y se procederá a una valoración en las mismas condiciones que el pretest, si bien prolongado durante 60´ para contrastar los procesos de recuperación y reactivación post-ejercicio. Finalizada esta medición se efectuará una valoración final de rigidez arterial. Como ya se indicó, CON tendrá el mismo contenido que las sesiones experimentales, si bien no serán efectuados los procesos de calentamiento, de indicadores de fatiga neuromuscular, ni la realización de los diferentes ejercicios. Todas las mediciones se realizarán con las participantes en estado postprandial (3 horas posteriores a la última ingesta de alimentos), solicitándoles evitar la ingesta de cafeína el día de medición y la realización de actividad física intensa en las 24 horas precedentes a la medición. Se solicitará a las participantes que mantengan sus hábitos de hidratación en las horas previas al ejercicio, si bien durante el mismo no se permitirá la ingesta de líquido al ser este un factor que puede incidir en algunos de los resultados, tales como los niveles de HPE. Debe indicarse que para la realización de esta fase del proyecto, el grupo cuenta con todo el equipamiento necesario, salvo la prensa de piernas horizontal y el medidor de velocidad de la onda del pulso (pOpmetre; Axelife. Saint Nicolas de Redon, Francia) necesario para estimar la rigidez arterial. Por ello en el presupuesto se incluyen estos dos equipos, así como el coste de fungibles de los equipos disponibles.

## Cronograma y fecha prevista de finalización. Distribución de las tareas entre los miembros del equipo investigador

El periodo de ejecución es de 3 años. Estimando la autorización por parte del CEI en el mes de septiembre de 2022, el proyecto se extendería aproximadamente desde noviembre de 2022 a noviembre de 2025. A continuación, se detalla la planificación del proyecto a lo largo de los meses de ejecución, incluidas las acciones de transferencia.

Reclutamiento

- *Hitos*: **1)** **Diseño e implementación de acciones de captación de usuarias de las instalaciones deportivas potencialmente candidatas para participar en el estudio**. RESPONSABLES: Eliseo Iglesias Soler y Xurxo Dopico; MIEMBROS DE EQUIPO DE INVESTIGACIÓN Y DEL EQUIPO DE TRABAJO PARTICIPANTES: Xurxo Dopico, Eduardo Carballeira, Jessica Rial, María Rúa Alonso, Enrique Fraga Germade. MESES DEL PROYECTO: 1º y 2º. **2) Consulta médica inicial, aplicación de criterios de inclusión y exclusión y configuración final de la muestra.** RESPONSABLE: Manuel Giráldez García. MIEMBROS DE EQUIPO DE INVESTIGACIÓN Y EQUIPO DE TRABAJO PARTICIPANTES: Ana Martínez Cagiao (enfermera), Jessica Rial, María Rúa Alonso, Lucía Vila Barrios (enfermera).

Fase experimental

- *Hitos*: **1) Evaluación pretest (sesiones 2 a 4).** RESPONSABLES: Eliseo Iglesias Soler; Manuel Giráldez García. MIEMBROS DE EQUIPO DE INVESTIGACIÓN Y DEL EQUIPO DE TRABAJO PARTICIPANTES: Alexis Padrón Cabo, Gonzalo Márquez, Xurxo Dopico Calvo, Ana Martínez Cagiao (enfermera), Manuel Giráldez García, Eduardo Carballeira, Jessica Rial, María Rúa Alonso, Adrián Varela Sanz, Juan Fariñas, Iván Nine Sieira, Enrique Fraga Germade. DURACIÓN: 4 semanas. **2) Realización de la recogida de datos en sesiones experimentales (sesiones 5 a 8)**. RESPONSABLES: Eliseo Iglesias Soler; Gonzalo Márquez. MIEMBROS DE EQUIPO DE INVESTIGACIÓN Y DEL EQUIPO DE TRABAJO PARTICIPANTES: Manuel Giráldez García, Alexis Padrón Cabo, Jessica Rial, María Rúa Alonso, Ana Martínez Cagiao, Adrián Varela Sanz, Juan Fariñas, Eduardo Carballeira, Iván Nine Sieira, Enrique Fraga Germade, Lucía Vila Barrios.

Fase de análisis de los resultados del estudio

- *Hitos*: **1) Análisis previo.** RESPONSABLE: Eliseo Iglesias Soler, Gonzalo Márquez. MIEMBROS DE EQUIPO DE INVESTIGACIÓN Y DEL EQUIPO DE TRABAJO: Eduardo Carballeira, Jessica Rial, María Rúa Alonso, Adrián Varela Sanz, Iván Nine Sieira, Pablo Díaz Brage. DURACIÓN: 6 semanas. MESES DEL PROYECTOS: 9º y 10º. **2) Inicio de redacción de comunicaciones a congresos y artículo con resultados del estudio**. RESPONSABLES: Eliseo Iglesias Soler, Manuel Giráldez García; MIEMBROS DE EQUIPO DE INVESTIGACIÓN Y DEL EQUIPO DE TRABAJO: Gonzalo Márquez, Xurxo Dopico Calvo, Alexis Padrón Cabo, Jessica Rial, María Rúa Alonso, Adrián Varela Sanz, Juan Fariñas, Eduardo Carballeira, Iván Nine Sieira.

Actividades de transferencia del estudio.

- *Hito*. **Acciones de transferencia y divulgación**. RESPONSABLES: Eliseo Iglesias Soler. MIEMBROS DE EQUIPO DE INVESTIGACIÓN Y DEL EQUIPO DE TRABAJO PARTICIPANTES: Gonzalo Márquez, Xurxo Dopico Calvo, Manuel Giráldez García, Jessica Rial, María Rúa Alonso, Adrián Varela Sanz, Juan Fariñas, Eduardo Carballeira, Iván Nine Sieira.

Figura 1. Cronograma del proyecto.

##

## Plan de análisis estadístico

El análisis estadístico se llevará a cabo mediante los programas SPSS versión 27.0 (SPSS, IBM, Armonk, NY, USA) o mediante diferentes paquetes estadísticos (nparLD, lme4, rcompanion) para R (R software v4.1.2. R Foundation, Vienna, Austria). Además de estadística descriptiva, con medidas de tendencia central y dispersión, se aplicarán diferentes procedimientos de estadística inferencias seleccionados adecuadamente en función de las variables analizadas, la estructura del diseño o las propiedades estadísticas de las variables. Señalamos los principales recursos a emplear:

- ANOVA factorial de medidas repetidas (2 factores de medidas repetidas, o factor de medidas repetidas y factor inter-sujeto).
- ANCOVA con medidas repetidas, con valoración de base como covariable.
- Modelos lineales mixtos, considerando efectos fijos y aleatorios. Este procedimiento además permitirá analizar en aquellas situaciones donde pudiese acontecer la pérdida de algún registro.
- Análisis de correlación lineal.
- Análisis de regresión lineal y no lineal.
- Cálculo de tamaños del efecto: eta cuadrado parcial, g de Hedge etc.
- Estadística no paramétrica. En caso de incumplimiento de alguna de las asunciones de los procedimientos paramétricos, incluso tras transformación de variables (por ejemplo, logarítmica) se emplearán procedimientos no paramétricos alternativos. Citamos entre ellos:
- ANOVA no paramétrico.
- Correlación no paramétrica (Spearman).
- Tamaño del efecto no paramétrico: Correlación biserial de rangos (Rank Biserial Correlation).
- Tests de Friedman, Kruskall-Wallis, Wilcoxon, Mann-Whithney.

# Aspectos ético-legales

## Cumplimiento de Normas de Buena Práctica Clínica, Declaración de Helsinki, Convenio de Oviedo, así como normativa de protección de datos, manejo de historia clínica y otra normativa de aplicación según el tipo de estudio

El presente proyecto implica la investigación en humanos y la utilización de muestras biológicas de origen humano y por ello se contemplará el respeto a las normas éticas y legales aplicables, en particular la Ley 14/2007, la Declaración de Helsinki y el Convenio de Oviedo y seguirá las Normas de buena práctica en investigación en seres humanos en su realización. Asimismo, el documento de consentimiento informado que se suministrará a las participantes tendrá en cuenta la siguiente normativa: Ley 3/2001, Ley 3/2005, Ley 41/2002, Decreto 29/2009 (Galicia), Decreto 164/2013 (Galicia) e Instrucción 6/2007 (Galicia).

## Modelos de consentimiento informado.

Se adjunta a la solicitud el modelo de consentimiento informado que se ha elaborado ha tenido en cuenta la siguiente normativa: Ley 3/2001, Ley 3/2005, Ley 41/2002, Decreto 29/2009 (Galicia), Decreto 164/2013 (Galicia) e Instrucción 6/2007 (Galicia).

# Memoria económica y fuente de financiación

El presente proyecto ha sido seleccionado dentro de la convocatoria Proyectos de Generación de Conocimiento 2021 del Ministerio de Ciencia e innovación (Código PID2021-124277OB-I00; <https://www.aei.gob.es/convocatorias/buscador-convocatorias/proyectos-generacion-conocimiento-2021/publicaciones>).

Presentamos a continuación una captura de la financiación asignada:


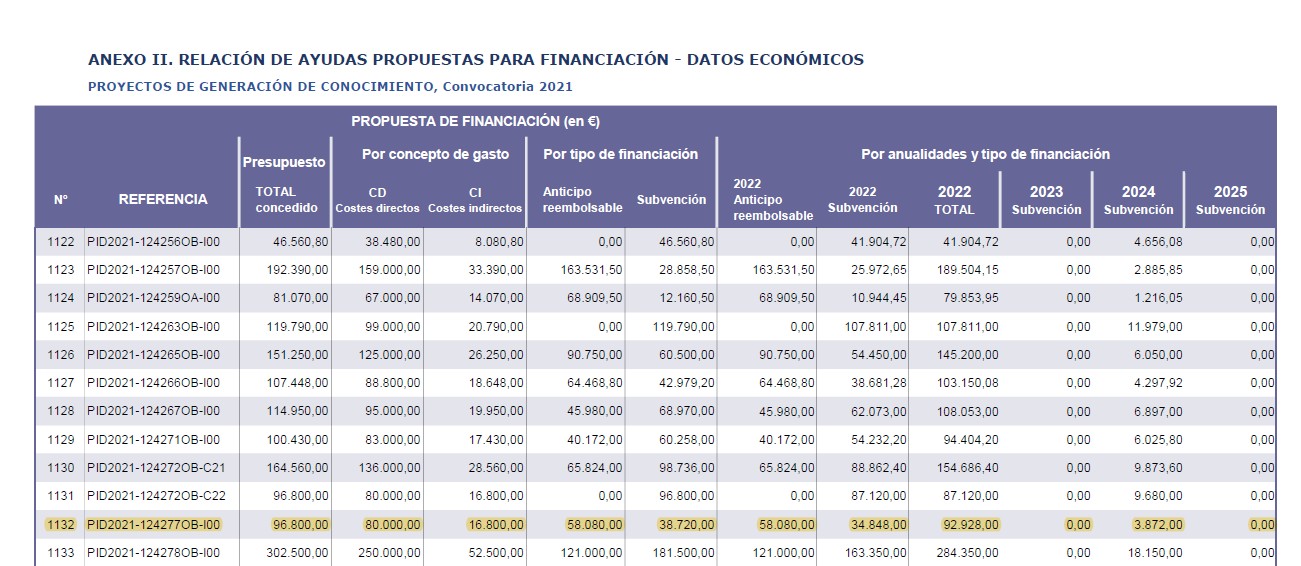


# Bibliografía

1. Murray CJL, Aravkin AY, Zheng P, Abbafati C, Abbas KM, Abbasi-Kangevari M, et al. Global burden of 87 risk factors in 204 countries and territories, 1990–2019: a systematic analysis for the Global Burden of Disease Study 2019. Lancet. 2020 Oct 17;396(10258):1223–49.
2. Grau M, Elosua R, Cabrera de León A, Guembe MJ, Baena-Díez JM, Vega Alonso T, et al. Factores de riesgo cardiovascular en España en la primera década del siglo XXI: análisis agrupado con datos individuales de 11 estudios de base poblacional, estudio DARIOS. Rev Española Cardiol. 2011 Apr;64(4):295–304.
3. Menéndez E, Delgado E, Fernández-Vega F, Prieto MA, Bordiú E, Calle A, et al. Prevalence, Diagnosis, Treatment, and Control of Hypertension in Spain. Results of the Di@bet.es Study. Rev Española Cardiol. 2016 Jun;69(6):572–8.
4. Virani SS, Alonso A, Benjamin EJ, Bittencourt MS, Callaway CW, Carson AP, et al. Heart Disease and Stroke Statistics—2020 Update: A Report From the American Heart Association. Circulation. 2020 Mar 3;141(9).
5. Benjamin EJ, Muntner P, Alonso A, Bittencourt MS, Callaway CW, Carson AP, et al. Heart Disease and Stroke Statistics-2019 Update: A Report From the American Heart Association. Circulation. 2019 Mar;139(10):e56–528.
6. Mattioli AV, Sciomer S, Moscucci F, Maiello M, Cugusi L, Gallina S, et al. Cardiovascular prevention in women: A narrative review from the Italian Society of Cardiology working groups on “Cardiovascular Prevention, Hypertension and peripheral circulation” and on “Women Disease.” J Cardiovasc Med. 2019 Sep;20(9):575–83.
7. Rosano GMC, Vitale C, Marazzi G, Volterrani M. Menopause and cardiovascular disease: the evidence. Climacteric. 2007 Jan;10(sup1):19–24.
8. Perez CC. Invisible Women: Exposing Data Bias in a World Designed for Men. Random House; 2019.
9. Costello JT, Bieuzen F, Bleakley CM. Where are all the female participants in Sports and Exercise Medicine research? Eur J Sport Sci. 2014 Nov 17;14(8):847–51.
10. Vitale C, Fini M, Spoletini I, Lainscak M, Seferovic P, Rosano GM. Under-representation of elderly and women in clinical trials. Int J Cardiol. 2017 Apr;232:216–21.
11. Börjesson M, Onerup A, Lundqvist S, Dahlöf B, Borjesson M, Onerup A, et al. Physical activity and exercise lower blood pressure in individuals with hypertension: Narrative review of 27 RCTs. Br J Sports Med. 2016 Mar;50(6):356–61.
12. Brook RD, Appel LJ, Rubenfire M, Ogedegbe G, Bisognano JD, Elliott WJ, et al. Beyond Medications and Diet: Alternative Approaches to Lowering Blood Pressure. Hypertension. 2013 Jun;61(6):1360–83.
13. Arnett DK, Blumenthal RS, Albert MA, Buroker AB, Goldberger ZD, Hahn EJ, et al. 2019 ACC/AHA Guideline on the Primary Prevention of Cardiovascular Disease: A Report of the American College of Cardiology/American Heart Association Task Force on Clinical Practice Guidelines. Circulation. 2019 Sep;140(11):e596–646.
14. Pedersen BK, Saltin B. Exercise as medicine - Evidence for prescribing exercise as therapy in different chronic diseases. Scand J Med Sci Sport. 2015 Dec;25:1–72.
15. Lee D, Artero EG, Sui X, Blair SN. Mortality trends in the general population: the importance of cardiorespiratory fitness. J Psychopharmacol. 2010 Nov;24(4 Suppl):27–35.
16. Liu Y, Lee DC, Li Y, Zhu W, Zhang R, Sui X, et al. Associations of Resistance Exercise with Cardiovascular Disease Morbidity and Mortality. Med Sci Sports Exerc. 2019 Mar;51(3):499–508.
17. El-Kotob R, Ponzano M, Chaput JP, Janssen I, Kho ME, Poitras VJ, et al. Resistance training and health in adults: an overview of systematic reviews. Appl Physiol Nutr Metab. 2020 Oct;45(10 (Suppl. 2)):S165–79.
18. Pollock ML, Franklin BA, Balady GJ, Chaitman BL, Fleg JL, Fletcher B, et al. Resistance exercise in individuals with and without cardiovascular disease: Benefits, rationale, safety, and prescription: An advisory from the Committee on Exercise, Rehabilitation, and Prevention, Council on Clinical Cardiology, American Heart Associati. Circulation. 2000 Feb;101(7):828–33.
19. Visseren FLJ, Mach F, Smulders YM, Carballo D, Koskinas KC, Bäck M, et al. 2021 ESC Guidelines on cardiovascular disease prevention in clinical practice. Eur Heart J. 2021 Sep 7;42(34):3227–337.
20. MacDonald H V., Johnson BT, Huedo-Medina TB, Livingston J, Forsyth KC, Kraemer WJ, et al. Dynamic resistance training as stand-alone antihypertensive lifestyle therapy: A meta-analysis. J Am Heart Assoc. 2016 Oct;5(10).
21. Cornelissen VA, Smart NA. Exercise training for blood pressure: a systematic review and meta-analysis. J Am Heart Assoc. 2013 Feb;2(1):e004473.
22. Cornelissen VA, Fagard RH, Coeckelberghs E, Vanhees L. Impact of resistance training on blood pressure and other cardiovascular risk factors: A meta-analysis of randomized, controlled trials. Hypertension. 2011 Nov;58(5):950–8.
23. Cornelissen VA, Fagard RH. Effect of resistance training on resting blood a pressure: A meta-analysis of randomized controlled trials. J Hypertens. 2005;23(2):251–9.
24. Kelley GA, Kelley KS. Progressive resistance exercise and resting blood pressure: A meta- analysis of randomized controlled trials. Hypertension. 2000;35(3):838–43.
25. Pescatello LS, Franklin BA, Fagard R, Farquhar WB, Kelley GA, Ray CA. Exercise and Hypertension. Med Sci Sport Exerc. 2004 Mar;36(3):533–53.
26. De Sá CA, Catani D, Cardoso AM, Da Silva Grigoletto ME, Battiston FG, Corralo VS. Resistance training affects the hemodynamic parameters of hypertensive and normotensive women differently, and regardless of performance improvement. J Exerc Sci Fit. 2020 Sep;18(3):122.
27. Cornelissen VA, Fagard RH, Coeckelberghs E, Vanhees L. Impact of Resistance Training on Blood Pressure and Other Cardiovascular Risk Factors. Hypertension. 2011 Nov;58(5):950–8.
28. Horsman HM, Peebles KC, Galletly DC, Tzeng YC. Cardiac baroreflex gain is frequency dependent: Insights from repeated sit-to-stand maneuvers and the modified Oxford method. Appl Physiol Nutr Metab. 2013 Jun;38(7):753–9.
29. Braith RW, Stewart KJ. Resistance exercise training: Its role in the prevention of cardiovascular disease. Vol. 113, Circulation. Lippincott Williams & Wilkins; 2006. p. 2642–50.
30. McCartney N, McKelvie RS, Martin J, Sale DG, MacDougall JD. Weight-training-induced attenuation of the circulatory response of older males to weight lifting. J Appl Physiol. 1993;74(3):1056–60.
31. Fecchio RY, Brito LC, Peçanha T, de Moraes Forjaz CL. Potential Mechanisms Behind the Blood Pressure–Lowering Effect of Dynamic Resistance Training. Vol. 23, Current Hypertension Reports. Curr Hypertens Rep; 2021.
32. Fagard RH, Cornelissen VA. Effect of exercise on blood pressure control in hypertensive patients. Eur J Cardiovasc Prev Rehabil. 2007 Feb;14(1):12–7.
33. Loaiza-Betancur AF, Chulvi-Medrano I, Díaz-López VA, Gómez-Tomás C. The effect of exercise training on blood pressure in menopause and postmenopausal women: A systematic review of randomized controlled trials. Vol. 149, Maturitas. Elsevier; 2021. p. 40–55.
34. Kenney MJ, Seals DR. Postexercise hypotension: Key features, mechanisms, and clinical significance. Hypertension. 1993 Nov;22(5):653–64.
35. Marçal IR, Goessler KF, Buys R, Casonatto J, Ciolac EG, Cornelissen VA. Post-exercise Hypotension Following a Single Bout of High Intensity Interval Exercise vs. a Single Bout of Moderate Intensity Continuous Exercise in Adults With or Without Hypertension: A Systematic Review and Meta-Analysis of Randomized Clinical Trials. Front Physiol. 2021 Jun;12.
36. Brito LC, Queiroz ACC, Forjaz CLM. Influence of population and exercise protocol characteristics on hemodynamic determinants of post-aerobic exercise hypotension. Brazilian J Med Biol Res [Internet]. 2014 Aug;47(8):626–36.
37. Teixeira L, Ritti-Dias RM, Tinucci T, Mion Jr. D, De Moraes Forjaz CL, Mion Júnior D, et al. Post-concurrent exercise hemodynamics and cardiac autonomic modulation. Eur J Appl Physiol. 2011 Sep;111(9):2069–78.
38. Casonatto J, Goessler KF, Cornelissen VA, Cardoso JR, Polito MD. The blood pressure-lowering effect of a single bout of resistance exercise: A systematic review and meta-analysis of randomised controlled trials. Eur J Prev Cardiol. 2016 Nov;23(16):1700–14.
39. Rúa-Alonso M, Mayo X, Mota J, Kingsley JD, Iglesias-Soler E. A short set configuration attenuates the cardiac parasympathetic withdrawal after a whole-body resistance training session. Eur J Appl Physiol. 2020;120(8):1905–19.
40. Río-Rodríguez D, Iglesias-Soler E, Fernández Del Olmo M. Set Configuration in Resistance Exercise: Muscle Fatigue and Cardiovascular Effects. PLoS One. 2016;11(3):e0151163.
41. Mayo X, Iglesias-Soler E, Carballeira-Fernández E, Fernández-Del-Olmo M. A shorter set reduces the loss of cardiac autonomic and baroreflex control after resistance exercise. Eur J Sport Sci. 2015;16(8):996–1004.
42. Iglesias-Soler E, Boullosa DA, Carballeira E, Sánchez-Otero T, Mayo X, Castro-Gacio X, et al. Effect of set configuration on hemodynamics and cardiac autonomic modulation after high-intensity squat exercise. Clin Physiol Funct Imaging. 2015 Jul;35(4):250–7.
43. Mayo X, Iglesias-Soler E, Fariñas-Rodríguez J, Fernández-Del-Olmo M, Kingsley JD. Exercise Type Affects Cardiac Vagal Autonomic Recovery After a Resistance Training Session. J strength Cond Res. 2016 Sep;30(9):2565–73.
44. Mayo X, Iglesias-Soler E, Kingsley JD, Dopico X. Interrepetition Rest Set Lacks the V-Shape Systolic Pressure Response Advantage during Resistance Exercise. Sports. 2017 Dec;5(4):90.
45. Paz GA, Iglesias-Soler E, Willardson JM, Maia M de F, Miranda H. Postexercise Hypotension and Heart Rate Variability Responses Subsequent to Traditional, Paired Set, and Superset Resistance Training Methods. J strength Cond Res. 2019 Sep;33(9):2433–42.
46. Mayo X, Iglesias-Soler E, Fustes-Piñeiro S, González-Hernández R. The effect of set configuration and type of resistance exercise on recovery blood pressure. In: 4th International Conference on Human Performance Development through Strength and Conditioning, NSCA 2014. Murcia, Spain; 2014.
47. MacDougall JD, Tuxen D, Sale DG, Moroz JR, Sutton JR. Arterial blood pressure response to heavy resistance exercise. J Appl Physiol. 1985 Mar;58(3):785–90.
48. Mccartney N. Acute responses to resistance training and safety. Med Sci Sport Exerc. 1999 Jan;31(1):31–7.
49. Rosenwinkel ET, Bloomfield DM, Arwady MA, Goldsmith RL. Exercise and autonomic function in health and cardiovascular disease. Cardiol Clin. 2001;19(3):369–87.
50. Bjarnason-Wehrens B, Mayer-Berger W, Meister ER, Baum K, Hambrecht R, Gielen S. Recommendations for resistance exercise in cardiac rehabilitation. Recommendations of the German Federation for Cardiovascular Prevention and Rehabilitation. Eur J Prev Cardiol. 2004;11(4):352–61.
51. Gomides R, Dias R, Souza D, Costa L, Ortega K, Mion D, et al. Finger blood pressure during leg resistance exercise. Int J Sports Med. 2010 Aug;31(08):590–5.
52. de Sousa NM, Magosso RF, Dipp T, Plentz RD, Marson RA, Montagnolli AN, et al. Continuous blood pressure response at different intensities in leg press exercise. Eur J Prev Cardiol. 2014 Nov;21(11):1324–31.
53. Sale DG, Moroz DE, McKelvie RS, MacDougall JD, McCartney N. Effect of Training on the Blood Pressure Response to Weight Lifting. Can J Appl Physiol. 1994 Mar;19(1):60–74.
54. Falkel JE, Fleck SJ, Murray TF. Comparison of Central Hemodynamics Between Powerlifters and Bodybuilders During Resistance Exercise. J Strength Cond Res. 1992;6(1):24.
55. Nery S de S, Gomides RS, da Silva GV, Forjaz CL de M, Mion D, Tinucci T, et al. Intra-arterial blood pressure response in hypertensive subjects during low- and high-intensity resistance exercise. Clinics. 2010;65(3):271–7.
56. Lamotte M, Strulens G, Niset G, Van De Borne P. Influence of different resistive training modalities on blood pressure and heart rate responses of healthy subjects. Isokinet Exerc Sci. 2005 Jan;13(4):273–7.
57. Lamotte M, Fournier F, Vanissum A, Van De Borne P. Influence of rest period duration between successive muscular strength sets on acute modifications of blood pressure and heart rate in the healthy subject. Isokinet Exerc Sci. 2006;14(4):349–55.
58. Lamotte M, Niset G, van de Borne P. The effect of different intensity modalities of resistance training on beat-to-beat blood pressure in cardiac patients. Eur J Cardiovasc Prev Rehabil. 2005 Feb;12(1):12–7.
59. Lamotte M, Fleury F, Pirard M, Jamon A, Borne P van de. Acute cardiovascular response to resistance training during cardiac rehabilitation: effect of repetition speed and rest periods. Eur J Cardiovasc Prev Rehabil. 2010 Jun;17(3):329–36.
60. Williams MA, Haskell WL, Ades PA, Amsterdam EA, Bittner V, Franklin BA, et al. Resistance Exercise in Individuals With and Without Cardiovascular Disease: 2007 Update. Circulation. 2007 Jul 31;116(5):572–84.
61. Tufano JJ, Brown LE, Haff GG. Theoretical and Practical Aspects of Different Cluster Set Structures: A Systematic Review. Vol. 31, Journal of Strength and Conditioning Research. NSCA National Strength and Conditioning Association; 2017. p. 848–67.
62. Mayo X, Iglesias-Soler E, Fernández-Del-Olmo M. Effects of Set Configuration of Resistance Exercise on Perceived Exertion. Percept Mot Skills. 2014 Dec;119(3):825–37.
63. Mayo X, Iglesias-Soler E, Kingsley JD. Perceived Exertion Is Affected by the Submaximal Set Configuration Used in Resistance Exercise. J strength Cond Res. 2019 Feb;33(2):426–32.
64. Iglesias-Soler E, Boullosa DA, Dopico X, Carballeira E. Analysis of Factors That Influence the Maximum Number of Repetitions in Two Upper-Body Resistance Exercises: Curl Biceps and Bench Press. J Strength Cond Res. 2010 Jun;24(6):1566–72.
65. Iglesias-Soler E, Carballeira E, Sánchez-Otero T, Mayo X, Jiménez A, Chapman ML, et al. Acute effects of distribution of rest between repetitions. Int J Sports Med. 2012 May;33(05):351–8.
66. Fariñas J, Mayo X, Giraldez-García MA, Carballeira E, Fernandez-Del-Olmo M, Rial-Vázquez J, et al. Set Configuration in Strength Training Programs Modulates the Cross Education Phenomenon. J Strength Cond Res. 2019;(12):1.
67. Iglesias-Soler E, Carballeira E, Sanchez-Otero T, Mayo X, Jimenez a, Chapman ML. Acute Effects of Distribution of Rest between Repetitions. Int J Sports Med. 2012;33:351–8.
68. Iglesias-Soler E, Carballeira E, Sánchez-Otero T, Mayo X, Fernández-del-Olmo M. Performance of maximum number of repetitions with cluster-set configuration. Int J Sports Physiol Perform. 2014 Jul;9(4):637–42.
69. Rial-Vázquez J, Mayo X, Tufano JJJ, Fariñas J, Rúa-Alonso M, Iglesias-Soler E. Cluster vs. traditional training programmes: changes in the force–velocity relationship. Sport Biomech. 2020 Mar;5:1–19.
70. Baum K, Ruther T, Essfeld D. Reduction of Blood Pressure Response During Strength Training Through Intermittent Muscle Relaxations.pdf. Int J Sport Med. 2003;24(6):441–5.
71. Veloso Ú, Monteiro W, Farinatti P. Do continuous and intermittent exercises sets induce similar cardiovascular responses in the elderly women? Rev Bras Med do Esporte. 2003;9(2):85–90.
72. Coelho CW, Hamar D, de Araújo CGS. Physiological responses using 2 high-speed resistance training protocols. J strength Cond Res. 2003 May;17(2):334–7.
73. Ribeiro-Torres O, de Sousa AFM, Iglesias-Soler E, Fontes-Villalba M, Zouhal H, Carré F, et al. Lower Cardiovascular Stress during Resistance Training Performed with Inter-Repetition Rests in Elderly Coronary Patients. Medicina (B Aires). 2020 May;56(6):264.
74. Malik M. Heart rate variability: standards of measurement, physiological interpretation and clinical use. Task Force of the European Society of Cardiology and the North American Society of Pacing and Electrophysiology. Circulation. 1996 Mar;93(5):1043–65.
75. Parati G, Saul JP, Rienzo M Di, Mancia G. Spectral Analysis of Blood Pressure and Heart Rate Variability in Evaluating Cardiovascular Regulation. Hypertension. 1995;25(6):1276–86.
76. Stuckey MI, Tordi N, Mourot L, Gurr LJ, Rakobowchuk M, Millar PJ, et al. Autonomic recovery following sprint interval exercise. Scand J Med Sci Sports. 2012 Dec;22(6):756–63.
77. Marasingha-Arachchige SU, Rubio-Arias JÁ, Alcaraz PE, Chung LH. Factors that affect heart rate variability following acute resistance exercise: A systematic review and meta-analysis. J Sport Heal Sci. 2020 Nov;
78. Kingsley JD, Figueroa A. Acute and training effects of resistance exercise on heart rate variability. Clin Physiol Funct Imaging. 2014 May;36(3):179–87.
79. Kingsley JD, Tai YL, Marshall EM, Glasgow A, Oliveira R, Parks JC, et al. Autonomic modulation and baroreflex sensitivity after acute resistance exercise: responses between sexes. J Sports Med Phys Fitness. 2019;59(6):1036–44.
80. Machado MV, Barbosa T de PC, Chrispino TC, Junqueira das Neves F, Rodrigues GD, Soares PP da S, et al. Cardiovascular and Autonomic Responses after a Single Bout of Resistance Exercise in Men with Untreated Stage 2 Hypertension. Int J Hypertens. 2021 Mar;2021:6687948.
81. Heffernan KS, Collier SR, Kelly EE, Jae SY, Fernhall B. Arterial stiffness and baroreflex sensitivity following bouts of aerobic and resistance exercise. Int J Sports Med. 2007;28(3):197–203.
82. Mota MRMR, Pardono E, Lima LCJ, Arsa G, Bottaro M, Campbell CSG, et al. Effects of treadmill running and resistance exercises on lowering blood pressure during the daily work of hypertensive subjects. J strength Cond Res. 2009 Nov;23(8):2331–8.
83. Niemelä THT, Kiviniemi AMAAM, Hautala AJA, Salmi JAJ, Linnamo V, Tulppo MMP. Recovery pattern of baroreflex sensitivity after exercise. Med Sci Sports Exerc. 2008 May;40(5):864–70.
84. Albert CM, Mittleman MA, Chae CU, Lee IM, Hennekens CH, Manson JE. Triggering of sudden death from cardiac causes by vigorous exertion. N Engl J Med. 2000 Nov;343(19):1355–61.
85. Mittleman MA, Maclure M, Tofler GH, Sherwood JB, Goldberg RJ, Muller JE. Triggering of Acute Myocardial Infarction by Heavy Physical Exertion - Protection against Triggering by Regular Exertion. N Engl J Med. 1993 Dec;329(23):1677–83.
86. Fred HL. More on weightlifting injuries. Texas Hear Inst J. 2014 Aug;41(4):453–4.
87. Franklin BA, Thompson CPD, Al-Zaiti SS, Albert CM, Hivert M-FF, Levine BD, et al. Exercise-Related Acute Cardiovascular Events and Potential Deleterious Adaptations Following Long-Term Exercise Training: Placing the Risks Into Perspective–An Update: A Scientific Statement From the American Heart Association. Circulation. 2020 Mar;141(13):E705–36.
