# Supplementary material for: Acute cardiovascular responses of postmenopausal women to resistance training sessions differing in set configuration: A study protocol for a crossover trial
Source: PLoS One. 2024 Oct 14;19(10):e0311524. doi: 10.1371/journal.pone.0311524 (PMC11472946; doi:10.1371/journal.pone.0311524)
Supplement: S2 Protocol — (DOCX) [file pone.0311524.s004.docx]

**Project title**

Cardiovascular adaptations to resistance exercise: Effect of set configuration in postmenopausal normo and hypertensive women

- **Protocol Version:** Version 1 dated 14/07/2022
- **Promoter:** Eliseo Iglesias Soler
- **Principal Investigator:** Eliseo Iglesias Soler. Associate Professor, Department of Physical Education and Sports. Universidade da Coruña.
- **Collaborating Researchers:**
  - **Research Team:**
    - Manuel Giráldez García (Department of Physical Education and Sports. Universidade da Coruña)
    - Xurxo Dopico Calvo (Department of Physical Education and Sports. Universidade da Coruña)
    - Gonzalo Márquez Sánchez (Department of Physical Education and Sports. Universidade da Coruña)
    - Alexis Padrón Cabo (Department of Physical Education and Sports. Universidade da Coruña)
  - **Work Team:**
    - María Rúa Alonso (Department of Physical Education and Sports. Universidade da Coruña)
    - Jessica Rial Vázquez (Department of Physical Education and Sports. Universidade da Coruña)
    - Ana Martínez Cagiao (Universidade da Coruña)
    - Lucía Vila Barrios (SERVICIO GALLEGO DE SALUD)
    - Adrián Varela Sanz (Department of Physical Education and Sports. Universidade da Coruña)
    - Eduardo Carballeira Fernández (Department of Physical Education and Sports. Universidade da Coruña)
    - Iván Nine Sieira (Department of Physical Education and Sports. Universidade da Coruña)
    - Enrique Fraga Germade (Department of Physical Education and Sports. Universidade da Coruña)
    - Pablo Díaz Brage (Universidade da Coruña)

**INDEX**

[Abstract 3](#_Toc178593610)

[Background and Justification of the Project 3](#_Toc178593611)

[Hypotheses of the Study 7](#_Toc178593612)

[Objectives 7](#_Toc178593613)

[Study type: 7](#_Toc178593614)

[Material and methods 7](#_Toc178593615)

[Study setting 7](#_Toc178593616)

[Definition of study subjects 8](#_Toc178593617)

[Selection and withdrawal of subjects 8](#_Toc178593618)

[Recruitment of participants 8](#_Toc178593619)

[Assignment/Randomization procedure 8](#_Toc178593620)

[Sample size justification 8](#_Toc178593621)

[Main and secondary variables 9](#_Toc178593622)

[Description of the intervention 9](#_Toc178593623)

[Schedule and estimated competition date. Distribution of tasks among team members 11](#_Toc178593624)

[Statistical Analysis Plan 12](#_Toc178593625)

[Ethical-legal aspects 12](#_Toc178593626)

[Compliance with Good Clinical Practice Guidelines, the Declaration of Helsinki, the Oviedo Convention, as well as data protection regulations, management of medical records, and other applicable regulations depending on the type of study 12](#_Toc178593627)

[Informed consent model. 12](#_Toc178593628)

[Financial report and fundung source 12](#_Toc178593629)

[References 13](#_Toc178593630)

# Abstract

Menopause causes hormonal changes that affect the acute and chronic physiological responses to exercise in postmenopausal women. In this regard, studies on cardiovascular responses to resistance exercise in women are scarce, making it particularly important to identify load parameters that modulate these adaptations. Previous studies by our research group have found that the set configuration influences the cardiovascular stress involved, although these results were obtained in young, healthy subjects. Therefore, the main objective of this project is to extend this line of research and analyze the effect of the set configuration on the cardiovascular responses of postmenopausal women. Additionally, since previous studies have shown that an individual's blood pressure level can influence the impact of resistance training programs on cardiovascular changes, we aim to compare these modifications in normotensive and hypertensive postmenopausal women. The project is designed to compare the effects of three training sessions with the same volume, intensity, duration, and work-rest ratio, but differentiated by set configuration. For this purpose, a sample of 60 postmenopausal women (30 normotensive and 30 hypertensive) will be recruited from local sports facilities to randomly perform three experimental sessions and one control session. Before and after each session, hemodynamic responses (systolic, diastolic, and mean blood pressure), cardiac performance (heart rate, cardiac output, and stroke volume among others), cardiac and vascular autonomic modulation (heart rate variability and blood pressure variability), baroreflex effectiveness (baroreflex sensitivity), neuromuscular fatigue, arterial stiffness, and metabolic responses (lactacidemia, resting metabolic rate using indirect calorimetry) will be evaluated. This analysis will identify set configurations with the highest and lowest cardiovascular stress. This project will provide useful information to optimize the prescription of resistance exercise in postmenopausal women, by identifying effort structures that potentially allow for both reducing cardiovascular stress and preserving physiological adaptations.

# Background and Justification of the Project

Hypertension (HTN), or chronic elevation of blood pressure (BP), is one of the main risk factors for cardiovascular disease (CVD), responsible for an estimated 10.8 million deaths annually worldwide (1). In Spain, the prevalence of HTN is over 40% of the population and increases with age. While middle-aged individuals show a prevalence of 44%, it exceeds 75% in those over 60 years old (2,3). Specifically, in the female population, in addition to the traditional cardiovascular risk factors (4), there are specific factors after menopause (5,6): the withdrawal of estrogen is associated with changes in body fat distribution, reduced glucose tolerance, abnormal lipid levels, increased BP, heightened sympathetic tone, endothelial dysfunction, and vascular inflammation… (7). Consequently, although the prevalence of HTN among premenopausal women tends to be lower than that of men of similar age, it becomes higher in women after menopause (5), reaching the previously mentioned values (3). Additionally, hormonal changes in postmenopausal women increase the likelihood of developing osteoporosis, cancer, urinary and pelvic floor disorders, and dementia, among other conditions. The simultaneous effects of hormonal changes and aging post-menopause, coupled with the general underrepresentation of women in research (8,9,10), warrant greater attention to studying this population group.

Non-pharmacological treatment of HTN through lifestyle modification is the first therapeutic option, and reducing sedentary behavior along with implementing physical exercise programs has been shown to be highly effective (11–14). Due to its relevance in improving the cardiovascular system and the clear association between good aerobic fitness and reduced relative risk of CVD mortality (15), attention has primarily focused on promoting and researching aerobic or cardiorespiratory work. However, emerging evidence on the benefits derived from strength training adaptations, at structural (prevention of sarcopenia or osteoporosis), functional (improved mobility, increased daily physical activity, fall risk reduction), cognitive function and mental health, metabolic (decreased glycated hemoglobin, increased basal metabolism, increased lean body mass, improved lipid profiles), or cardiovascular levels (reduced BP, improved autonomic control), as well as mortality and morbidity reduction (16–18), has led to the development of the muscular component through strength-oriented exercises gaining relevance in physical exercise programs for health. In fact, very recently, the European Society of Cardiology has included strength training among its recommendations for physical activity and exercise (19).

The effect on reducing BP of programs aimed at developing muscular strength through dynamic exercises is significant (20–26), although smaller in magnitude than those obtained through other intervention models such as aerobic exercise or low-intensity isometric training (21). Successive meta-analyses have quantified the average reduction in both systolic (SBP) and diastolic blood pressure (DBP) by around 3 mmHg through dynamic exercise programs with overload, values that, although modest, can epidemiologically result in a decrease of between 5 and 9% in cardiac morbidity, between 8 and 14% in the risk of stroke, or a 4% reduction in mortality risk (12,27–29).

Furthermore, it has been shown that improving strength through resistance training attenuates the hemodynamic response to handling absolute load magnitudes (30), suggesting it may have an effect on improving muscle performance and consequent reduction in cardiac demands during daily activities (18). Dynamic strength training also promotes vascular adaptations by improving vascular conductance and endothelial function, which could play a role in the blood pressure-reducing effect of this type of training (31). Despite this evidence, various meta-analyses addressing the impact of strength training on baseline BP levels agree on the need to deepen our understanding of this phenomenon due to the limited number of studies currently available, particularly regarding those conducted in hypertensive populations (12,27,29,31,32). For example, in the meta-analysis conducted by Cornelissen et al. (21), it was only possible to include 29 groups corresponding to dynamic resistance exercise programs, compared to the 105 groups considered for the analysis of aerobic exercise program effects. On the other hand, the effect of strength training in postmenopausal women has shown positive effects for BP reduction (33). However, as observed in the recent review by Loaiza-Betancur et al. (33), specific studies on cardiovascular adaptations to strength training in postmenopausal women are scarce, even more so in women with hypertensive conditions; most studies include sedentary women, and to date, only one study has investigated the effects of physical exercise in physically active postmenopausal women. This reflects a certain degree of uncertainty about adaptations to strength training in this specific population group, which, in principle, could also benefit from it.

A comprehensive evaluation of the cardiovascular effect of strength development programs requires considering both chronic adaptations and acute hemodynamic responses. In this regard, the phenomenon of Post-Exercise Hypotension (PEH), which is the decrease in BP to levels lower than those measured before the start of the session, has been observed following both aerobic activities and strength development routines, particularly in hypertensive populations (34–38). This makes physical exercise a valuable tool for the non-pharmacological control of hypertension (12–14).

As will be noted later, our group has developed a line of research exploring cardiovascular responses and adaptations to resistance exercise, analyzing, among other issues, the factors influencing BP in a young, healthy, active, and predominantly male population (39–46). Therefore, there is a need to extend our findings to other population profiles, potentially different in terms of their physiological responses and adaptations, such as active postmenopausal women.

Despite the acute and chronic benefits derived from strength development programs, there is some caution when implementing them in certain populations because working with overloads entails a pronounced hemodynamic response during its execution (47,48), which could be a risk factor that needs to be considered in patients with some type of heart disease (49,50). In regular practice, resistance training consists of one or more sets of repetitions of one or several exercises. During the execution of successive repetitions in the same set, there is a progressive loss of mechanical performance (power, speed, etc.) and, simultaneously, a continuous increase in BP, both systolic (SBP) and diastolic (DBP), reaching its peak value at the moment of muscle failure (47,48,51,52). Simultaneously, heart rate (HR) also increases, and consequently, the Double Product (DP = HR x SBP) rises (47,48). Although the increase in SBP in an isolated contraction is dependent on its intensity (53), it has been shown that the duration of the set is the main factor influencing this elevation (54–59). Thus, low-intensity sets with a high number of repetitions produce higher cardiovascular responses compared to higher-intensity sets with fewer repetitions (54–59). Despite this, the recommendation to perform exercises with light weights and numerous repetitions still predominates in the realm of prescribing resistance exercise for people with cardiovascular diseases (18,60). Given that the level of strength development significantly depends on the mechanical stimulus generated during the exercises (force, power, muscle tension), and that the temporal extension of work sets can entail a more pronounced hemodynamic response, it is possible to deduce that the currently recommended guidelines for subjects with cardiovascular pathology may have limited effects on strength improvement and, at the same time, cause greater cardiovascular stress during the practice of resistance exercise, with all the risks this entails.

Therefore, there is a need to design intervention strategies that combine an increase in the quality of the mechanical stimulus with a reduction in cardiovascular responses during exercise. In this regard, an alternative to the traditional exercise model—which consists of performing continuous repetitions until near muscle failure—is emerging. This alternative set design is based on modifying the work and rest periods, breaking the set into small groups of repetitions. This type of training has been given different names: Cluster Training, Inter-repetition rest training, or Intra-Set Rest Loading (61). In this respect, various studies by our group have shown that shorter, cluster set configurations lead to a reduction in perceived exertion (62,63) and improved mechanical performance during the session in terms of speed and power (64–69). The cluster set configuration would involve less mechanical compression time of the muscle vessels or less time and intensity performing the Valsalva maneuver, factors responsible for the hemodynamic response during resistance exercise (48). Previous studies have demonstrated that incorporating small relaxation pauses between repetitions is an effective strategy for reducing BP increases during resistance exercise (70–72). However, these studies did not equalize the work-to-rest ratio, so the sessions with rests between repetitions accumulated more recovery time and thus increased the duration of the sessions. These inequalities between configurations could have influenced the results. Therefore, it is necessary to evaluate the effect of the set configuration (cluster-type vs. traditional training) by isolating this factor from others that could affect the results. This means evaluating the effect of the set configuration requires that other load factors (volume, intensity, total recovery time) remain stable between conditions. Our group has worked on this line of research in recent years, observing that, compared to a traditional configuration, relatively short set configurations (i.e., a set structure where around 30-40% of maximum repetitions are performed) are the ones that attenuate the BP response (44). However, these assessments were obtained from a sample of young, healthy, and physically active individuals (students of Physical Activity and Sports Sciences) and predominantly male. In a recent study with coronary patients, in which the PI of this project participated (73), this attenuation of cardiovascular stress with short set configurations was also verified, although in this case, the sample was exclusively male. Therefore, it would be appropriate to implement this type of analysis in the female population, particularly in postmenopausal women diagnosed with hypertension. The evidence suggests that this population group could greatly benefit from shorter set structures, as they would provide the benefits of strength training with lower cardiovascular risk. Additionally, considering the adverse responses that can be observed during menopause (fatigue, tiredness, etc.), these protocols with short sets generate lower perceptions of effort during exercise and could favor adherence to training programs.

Hemodynamic responses to exercise are linked to variations in cardiac and vascular control largely mediated by the Autonomic Nervous System (ANS). Currently, cardiac autonomic control can be evaluated non-invasively through the analysis of factors such as heart rate variability (HRV) or heart rate complexity (HRC), while sympathetic vascular tone can be estimated by analyzing blood pressure variability (BPV) (74,75). Complementarily, the combined beat-to-beat analysis of SBP and HR allows for the assessment of baroreflex sensitivity (BRS), understood as the efficacy of the baroreflex in changing HR in response to changes in SBP (76). Few studies have analyzed the impact of resistance exercise on cardiac autonomic control and BRS. Previous works have shown a transient loss of cardiac autonomic control (77,78) and a decrease in BRS immediately following a session of resistance exercises (79–83). These reductions in both variables create a situation of vulnerability, as the risk of certain cardiovascular events is associated with low levels of HRV and BRS, especially in populations with cardiovascular risk (84–87).

Our research group has been a pioneer in analyzing the effect of set configuration on acute changes in HRV (39–45). The results of these studies show an attenuation of the loss of autonomic control when using short, cluster set configurations compared to traditional structures close to muscle failure. This suggests the use of set configuration as a regulatory element for changes in cardiac autonomic modulation induced by resistance exercise. Again, all these studies were conducted with young, healthy, and male samples, making it appropriate to confirm their findings in other population profiles, such as the one targeted by this project. It has also been suggested that the decrease in BRS following resistance exercise may be conditioned by a reprogramming of the reflex due to the BP peaks reached during exercise (81). Therefore, it is possible to hypothesize that training designs that generate a lower hemodynamic response will also result in less deterioration of BRS, consequently making them safer protocols with lower cardiovascular risk. This issue has also been addressed in the previously mentioned studies, which observed a smaller decrease in BRS after performing exercise routines with short, cluster-type set configurations compared to more conventional structures. Thus, our studies also seem to indicate less post-exercise BRS deterioration in young, healthy populations after working with cluster-type structures. All this allows us to conclude that cluster or short set configurations are effort structures that enable the improvement of mechanical muscle stimulation, the reduction of hemodynamic stress during the session, and the attenuation of the acute loss of parasympathetic and baroreflex modulation, making them training strategies with a more positive benefit-risk balance. However, as mentioned previously, it is not possible to extrapolate these conclusions to other population groups beyond young, healthy, and physically active individuals. Older individuals and those with cardiovascular pathology are the groups that could benefit most from the advantages of these types of structures. A clear example, considering the points previously mentioned and the lack of sufficient evidence, would be postmenopausal women with and without hypertension. Both factors (menopause and hypertension) create a differentiated physiological profile where the negative effects of hormonal changes and aging coexist. This highlights this population group as candidates for research to identify which strength training models could provide the greatest health and quality of life benefits with the lowest associated cardiovascular risk. Such research would make physical exercise a safe non-pharmacological treatment for managing hypertension and other specific consequences associated with menopause (osteoporosis, sarcopenia, etc.).

# Hypotheses of the Study

1. Based on previous findings from our group, we hypothesize an attenuation of hemodynamic responses and deterioration of autonomic and baroreflex cardiac control following resistance exercise with short set configurations compared to those induced by more conventional configurations.
2. Based on previous studies indicating a greater reduction in BP in hypertensive individuals and after long configurations close to muscle failure (43), we expect a hypotensive effect of resistance exercise, especially following longer, more fatiguing configurations, and particularly in the sample of hypertensive women.

# Objectives

*GENERAL OBJECTIVES*

- Evaluate the effect of set configuration during resistance exercise on acute hemodynamic and metabolic responses, as well as on regulatory mechanisms (cardiac autonomic control, sympathetic vasomotor tone) in active postmenopausal women with and without hypertension.
- Contrast acute hemodynamic responses between normotensive and hypertensive postmenopausal women.

*SPECIFIC OBJECTIVES*

- Evaluate the effect of set configuration on acute changes in hemodynamics, resting energy expenditure, and cardiac and vascular autonomic control in normotensive and hypertensive postmenopausal women.
- Determine the set configuration model that produces the least hemodynamic alteration and neural cardiac control disruption in normotensive and hypertensive postmenopausal women.
- Identify the set configuration with the least impact on the decrease in baroreflex sensitivity (BRS) post-exercise in normotensive and hypertensive postmenopausal women.
- Assess the association between hemodynamic changes during exercise and acute modifications in autonomic and baroreflex cardiac control in normotensive and hypertensive postmenopausal women.
- Identify the resistance exercise configuration that optimizes the post-exercise hypotension phenomenon in normotensive and hypertensive postmenopausal women.
- Contrast the cardiovascular impact of different set configurations in resistance exercise between normotensive and hypertensive postmenopausal women.

# Study type:

- Cross-over design with repeated measures, as each subject in the sample will go through all experimental conditions.

# Material and methods

## Study setting

This project falls within the field of Physical Activity and Sports Sciences.

## Definition of study subjects

The sample will consist of postmenopausal women who are users of two facilities located in the influence area of the city of A Coruña: Termaria Casa del Agua (<https://termaria.es/>) and DOS Acea da Ma (<https://www.dosdeporte.es/inicio-acea-de-ama/>). As reflected in the PI's CV, both facilities have hosted some of the research group's transfer projects and have active agreements with the University of A Coruña. Additionally, the research group has contacted the company responsible for managing sports centers in the Oleiros area, near the Faculty where the present project will be carried out (Aqualia: <http://www.centrosdeportivosoleiros.es/oleiros/>) to recruit participants.

## Selection and withdrawal of subjects

All participants in the sample will undergo a pre-participation medical consultation to assess potential absolute and relative contraindications for exercise and inclusion/exclusion criteria. This selection process will be supervised by Dr. Manuel Giráldez García, a member of the research team and a specialist in Sports Medicine. The inclusion criteria will be as follows: for all women, having been postmenopausal for at least 5 years (counted after 12 consecutive months without menstruation), aged between 55 and 64 years, physically active (150-300 min/week of light-intensity or 75-150 min/week of moderate-to-vigorous-intensity exercise), having no more than 3 cardiovascular risk factors, being asymptomatic and without known cardiovascular, metabolic, or renal disease, and having a valid EU COVID certificate; for women with hypertension, in addition to the above, being diagnosed with well-controlled grade I hypertension with no more than one medication and low or moderate cardiovascular risk (*Hypertension*. 2020;75:1334-1357. DOI: 10.1161/HYPERTENSIONAHA.120.15026.). The exclusion criteria will be: having any other grade of hypertension, needing more than one medication to control hypertension or using any other medication that interferes with cardiovascular responses to exercise, being or having been on hormone replacement therapy, and presenting hypertensive response to exercise. Each participant must sign the corresponding Informed Consent Form (ICF), which will detail all the study characteristics and will follow the model suggested by the Galician Network of Research Ethics Committees (<https://acis.sergas.es/cartafol/05-Modelos-de-documentos>). As stated in the mentioned document, all participants will have the right to withdraw from the experiment if they wish to do so.

## Recruitment of participants

The sample will be recruited by members of the research team through the placement of posters, registration sheets, and QR codes at the reception and bulletin boards of the facilities participating in the project. Additionally, a project profile will be created on major social media platforms (Twitter/X, Instagram, Facebook) outlining the inclusion criteria, and it will be announced through the research group's website.

## Assignment/Randomization procedure

This is a crossover repeated measures design, in which the order of the different experimental conditions will be randomized.

## Sample size justification

A sample size calculation has been conducted to achieve a statistical power of 80%, at a significance level of 0.05, to detect a small effect size (f=0.12) for the interaction between a within-subject factor (3 configurations and control session) and a between-subject factor (normo and hypertensive groups), assuming a correlation between repeated measures of 0.75. The result of the calculation, using the G*Power v3.1.9.7 software, is a total sample size of 50 women (25 each group). We aim for a total sample of 60 postmenopausal women: 30 normo and 30 hypertensives.

## Main and secondary variables

| *Main variables:* | *Secondary variables:* |
| --- | --- |
| - Systolic, diastolic, and mean arterial blood pressure | - Age |
| - Baroreflex sensitivity | - Height |
| - Heart rate variability | - Body mass |
| - Blood pressure variability | - Body composition |
| - Arterial stiffness | - Bone mineral density |
| - Resting oxygen consumption | - Years since menopause |
| - Cardiac output | - Hypertension treatment |
| - Stroke volume | - 12-Repetition maximum load |
| - Total peripheral resistance at rest | - Exercise execution speed |
| - Lactate level | - Resting electrocardiogram results |
| - Perceived exertion | - Maximal oxygen consumption |

## Description of the intervention

*DESIGN*: Considering the objectives of the study, each participant in the sample will carry out, in a randomized sequence, three exercise sessions and one control session. The exercise session will consist of completing the same volume of work (repetitions x kilograms) in leg press, bench press, leg curl, and lat pulldown exercises, but responding to different structures regarding the set configuration. Thus, with a load of 12 maximum repetitions (12RM), an equivalent volume will be completed in each exercise, consisting of 3 sets of 12 repetitions in each exercise, varying the effort structure in each of the sessions: a session with 9 sets of 4 repetitions (4S), corresponding to 33% of effort intensity (4 out of 12 possible repetitions); a session with 6 sets of 6 repetitions (6S), corresponding to 50% effort intensity (6 out of 12 possible repetitions); and finally, a session with 4 sets of 9 repetitions (9S), corresponding to 75% effort intensity. To ensure an equivalent work/rest ratio between sessions, the recovery between sets will be 45, 72, and 120 seconds for 4S, 6S, and 9S, respectively. The rest between exercises will be 4 minutes for all sessions. This procedure ensures equality between sessions in terms of volume, load intensity, recovery time, and session duration, with the independent variable being the set configuration, i.e., the required effort intensity. Additionally, a control session (CON) will be conducted without exercise to provide a baseline reference for the hemodynamic and metabolic behavior of the sample over time. The structure for recording will be the same in 4S, 6S, and 9S: **i)** initial recording for 15 minutes at rest of hemodynamic, metabolic (resting oxygen consumption), indicators of cardiac autonomic modulation, cardiac performance, and measurement of arterial stiffness; **ii)** 10-minute warm-up phase; **iii)** neuromuscular assessment to control fatigue; **iv)** baseline lactate measurement; **v)** completion of the session with an approximate duration of 45 minutes; **vi)** post-exercise lactate measurement; **vii)** neuromuscular measurement after exercise; **viii)** measurement of arterial stiffness; **ix)** repetition of hemodynamic, metabolic, and cardiac autonomic modulation recording at rest for 60 minutes to assess the hypotensive effect of exercise; and **x)** final measurement of arterial stiffness. CON will consist of repeating the aforementioned structure, replacing the exercise routine with 45 minutes of rest. This phase of the project will be carried out in the facilities of the Faculty of Sport Sciences and Physical Education of the University of A Coruña.

*PROCEDURES:* Each subject will undergo a total of 8 assessment sessions: **Session 1**: Medical consultation (Manuel A. Giráldez García) to evaluate health status, potential cardiovascular risk factors, contraindications (absolute and relative), and inclusion/exclusion criteria. This consultation will include medical history (including provided previous medical reports), comprehensive physical examination, and complementary tests (with prior informed consent): resting electrocardiogram (ECG), resting spirometry, and ergospirometry with ECG, gas, and BP monitoring. **Session 2**: Anthropometric measurements (weight, height, body mass index, body composition by bioimpedance, skinfold and diameter measurements), bone mineral density using an ultrasound bone densitometer (Sonost 3000, Osteosys Corp., Korea), and familiarization with exercise performance. Exercises have been selected according to international recommendations regarding the use of multi-joint exercises involving large muscle groups. **Sessions 3 and 4**. Dedicated to determining, for each exercise, the load at which the subject can perform a maximum of 12 repetitions (12RM). During the procedure, execution speed will be recorded using the T-Force device to guide load adjustments based on loss of propulsive speed. The procedure will be conducted twice to determine the replicability of the results. A difference of more than 10% between the defined 12RM loads in each session will require a third assessment. Sessions will be separated by at least 72 hours of recovery. **Sessions 5-8**. Correspond to the randomized completion of the 4S, 6S, 9S, and CON sessions. The steps for each session are as follows: Once the subject arrives at the laboratory, they will be prepared for recording using the Task Force Monitor device (CNSystems, Graz, Austria) for ECG, beat-to-beat BP, and impedance cardiography. Additionally, indirect measurement of resting metabolic rate (oxygen consumption) will be conducted using the MetaMax 3B-R2 analyzer (Cortex, Leipzig, Germany). After calibrations, the recording will begin, with the subject lying supine on a stretcher in silence for 15 minutes. Afterward, arterial stiffness will be assessed using the finger-toe pulse wave velocity measurement technique. Next, the warm-up phase will start, consisting of 5 minutes of cycling on an ergometer at a moderate intensity (50-70% of their estimated maximum HR), 2 minutes of joint mobility, and performing 10 repetitions of each exercise at 70% of the load to be used in the session at a moderate execution speed. After 2 minutes of recovery, baseline lactate will be recorded, and the speed of movement for each exercise will be recorded using the T-Force transducer with a load equivalent to 70% of 12RM. This assessment will be used as an indicator of muscle fatigue. The exercise session will then begin, following the previously described guidelines. During this phase, in addition to measuring cardiovascular parameters, mechanical performance in each repetition will be recorded using the T-Force device, as well as perceived exertion at the end of each set using the OMNI-RES scale. One minute after completing the last repetition of the session, a new lactate measurement will be taken, the subject will be instructed to lie back on the stretcher, arterial stiffness will be measured, and cardiovascular recording will be prepared using the Task-Force Monitor device. A post-exercise assessment will then be conducted under the same conditions as the pretest but extended for 60 minutes to contrast recovery and post-exercise reactivation processes. After this measurement, a final assessment of arterial stiffness will be performed. As mentioned earlier, the CON session will have the same content as the experimental sessions, although warm-up processes, neuromuscular fatigue indicators, and exercise performance will not be conducted. All measurements will be taken with participants in a postprandial state (3 hours after the last meal), and they will be asked to avoid caffeine intake on the day of measurement and intense physical activity in the 24 hours preceding measurement. Participants will be asked to maintain their hydration habits in the hours leading up to exercise, although fluid intake will not be allowed during exercise as it may affect some results, such as lactate levels. It should be noted that for this phase of the project, the group has all the necessary equipment except for the horizontal leg press and the pulse wave velocity meter (pOpmetre; Axelife. Saint Nicolas de Redon, France) required to estimate arterial stiffness. Therefore, the budget includes these two pieces of equipment, as well as the cost of consumables for the available equipment.

## Schedule and estimated competition date. Distribution of tasks among team members

The execution period is 3 years. Assuming approval from the Research Ethics Committee in September 2022, the project would extend approximately from November 2022 to November 2025. Below is the project's planning throughout the execution months, including transfer actions.

Recruitment

- *Milestones*: **1)** **Design and implementation of user recruitment actions from sports facilities potentially eligible to participate in the study**. RESPONSIBLE: Eliseo Iglesias and Xurxo Dopico; RESEARCH AND WORK TEAM PARTICIPANTS: Xurxo Dopico, Eduardo Carballeira, Jessica Rial, María Rúa Alonso, Enrique Fraga Germade. **2) Initial medical consultation, application of inclusion and exclusion criteria, and final configuration of the sample.** RESPONSIBLE: Manuel Giráldez García. RESEARCH AND WORK TEAM PARTICIPANTS: Ana Martínez Cagiao (nurse), Jessica Rial, María Rúa Alonso, Lucía Vila Barrios (nurse).

Experimental phase

- *Milestones*: **1) Pretest evaluation (sessions 2 to 4).** RESPONSIBLE: Eliseo Iglesias Soler; Manuel Giráldez García. RESEARCH AND WORK TEAM PARTICIPANTS: Alexis Padrón Cabo, Gonzalo Márquez, Xurxo Dopico Calvo, Ana Martínez Cagiao (enfermera), Manuel Giráldez García, Eduardo Carballeira, Jessica Rial, María Rúa Alonso, Adrián Varela Sanz, Juan Fariñas, Iván Nine Sieira, Enrique Fraga Germade. **2) Data collection during experimental sessions (sessions 5 to 8)**. RESPONSIBLE: Eliseo Iglesias Soler; Gonzalo Márquez. RESEARCH AND WORK TEAM PARTICIPANTS: Manuel Giráldez García, Alexis Padrón Cabo, Jessica Rial, María Rúa Alonso, Ana Martínez Cagiao, Adrián Varela Sanz, Juan Fariñas, Eduardo Carballeira, Iván Nine Sieira, Enrique Fraga Germade, Lucía Vila Barrios.

Analysis phase of study results

- *Milestones*: **1) Preliminary Analysis.** RESPONSIBLE: Eliseo Iglesias Soler, Gonzalo Márquez. RESEARCH AND WORK TEAM PARTICIPANTS: Eduardo Carballeira, Jessica Rial, María Rúa Alonso, Adrián Varela Sanz, Iván Nine Sieira, Pablo Díaz Brage. **2) Commencement of Drafting Communications for Conferences and Articles with Study Results**. RESPONSIBLE: Eliseo Iglesias Soler, Manuel Giráldez García; RESEARCH AND WORK TEAM PARTICIPANTS: Gonzalo Márquez, Xurxo Dopico Calvo, Alexis Padrón Cabo, Jessica Rial, María Rúa Alonso, Adrián Varela Sanz, Juan Fariñas, Eduardo Carballeira, Iván Nine Sieira.

Transfer Activities.

- *Milestones*. **Transfer and Dissemination Actions**. RESPONSIBLE: Eliseo Iglesias Soler. RESEARCH AND WORK TEAM PARTICIPANTS: Gonzalo Márquez, Xurxo Dopico Calvo, Manuel Giráldez García, Jessica Rial, María Rúa Alonso, Adrián Varela Sanz, Juan Fariñas, Eduardo Carballeira, Iván Nine Sieira.

Figure 1. Project timeline.

## Statistical Analysis Plan

The statistical analysis will be carried out using the SPSS software version 27.0 (SPSS, IBM, Armonk, NY, USA) or different statistical packages (nparLD, lme4, rcompanion) for R (R software v4.1.2. R Foundation, Vienna, Austria). In addition to descriptive statistics, including measures of central tendency and dispersion, different inferential statistical procedures will be applied, selected appropriately based on the analyzed variables, design structure, or statistical properties of the variables. We highlight the main resources to be used:

- Factorial repeated measures ANOVA (2 factors of repeated measures, or repeated measures factor and between-subjects factor).
- ANCOVA with repeated measures, with baseline assessment as covariate.
- Mixed linear models, considering fixed and random effects. This procedure will also allow analysis in situations where some data may be missing.
- Linear correlation analysis.
- Linear and nonlinear regression analysis.
- Calculation of effect sizes: partial eta squared, Hedge's g, etc.
- Nonparametric statistics. In case of violation of assumptions of parametric procedures, even after variable transformation (e.g., logarithmic), alternative nonparametric procedures will be used. These include:
- Nonparametric ANOVA.
- Nonparametric correlation (Spearman).
- Nonparametric effect size: Rank Biserial Correlation).
- Friedman, Kruskall-Wallis, Wilcoxon, Mann-Whithney tests.

# Ethical-legal aspects

## Compliance with Good Clinical Practice Guidelines, the Declaration of Helsinki, the Oviedo Convention, as well as data protection regulations, management of medical records, and other applicable regulations depending on the type of study

This project involves human research and the use of biological samples of human origin, and therefore it will comply with applicable ethical and legal standards, particularly Law 14/2007, the Declaration of Helsinki, and the Oviedo Convention. It will follow the guidelines of Good Practice in Human Research during its execution. Furthermore, the informed consent document provided to participants will consider the following regulations: Law 3/2001, Law 3/2005, Law 41/2002, Decree 29/2009 (Galicia), Decree 164/2013 (Galicia), and Instruction 6/2007 (Galicia).

## Informed consent model.

The informed consent model attached to the application has been developed taking into account the following regulations: Law 3/2001, Law 3/2005, Law 41/2002, Decree 29/2009 (Galicia), Decree 164/2013 (Galicia), and Instruction 6/2007 (Galicia).

# Financial report and fundung source

This project has been selected under the 2021 Knowledge Generation Projects call by the Ministry of Science and Innovation (Code PID2021-124277OB-I00; <https://www.aei.gob.es/convocatorias/buscador-convocatorias/proyectos-generacion-conocimiento-2021/publicaciones>).

Below is a snapshot of the assigned funding:


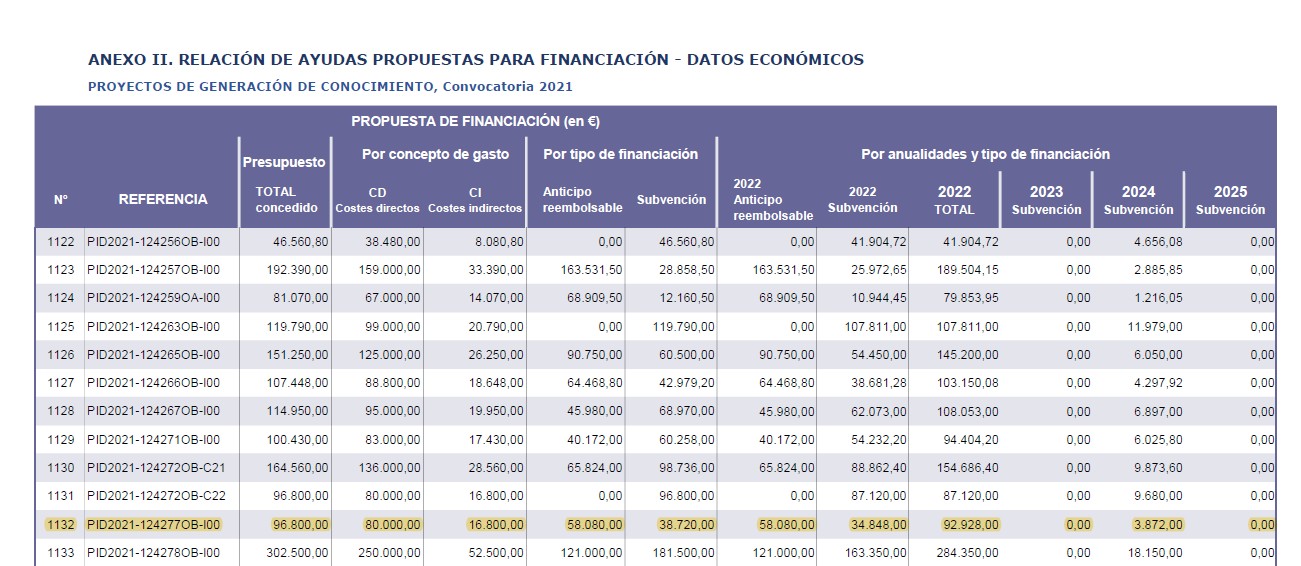


# References

1. Murray CJL, Aravkin AY, Zheng P, Abbafati C, Abbas KM, Abbasi-Kangevari M, et al. Global burden of 87 risk factors in 204 countries and territories, 1990–2019: a systematic analysis for the Global Burden of Disease Study 2019. Lancet. 2020 Oct 17;396(10258):1223–49.
2. Grau M, Elosua R, Cabrera de León A, Guembe MJ, Baena-Díez JM, Vega Alonso T, et al. Factores de riesgo cardiovascular en España en la primera década del siglo XXI: análisis agrupado con datos individuales de 11 estudios de base poblacional, estudio DARIOS. Rev Española Cardiol. 2011 Apr;64(4):295–304.
3. Menéndez E, Delgado E, Fernández-Vega F, Prieto MA, Bordiú E, Calle A, et al. Prevalence, Diagnosis, Treatment, and Control of Hypertension in Spain. Results of the Di@bet.es Study. Rev Española Cardiol. 2016 Jun;69(6):572–8.
4. Virani SS, Alonso A, Benjamin EJ, Bittencourt MS, Callaway CW, Carson AP, et al. Heart Disease and Stroke Statistics—2020 Update: A Report From the American Heart Association. Circulation. 2020 Mar 3;141(9).
5. Benjamin EJ, Muntner P, Alonso A, Bittencourt MS, Callaway CW, Carson AP, et al. Heart Disease and Stroke Statistics-2019 Update: A Report From the American Heart Association. Circulation. 2019 Mar;139(10):e56–528.
6. Mattioli AV, Sciomer S, Moscucci F, Maiello M, Cugusi L, Gallina S, et al. Cardiovascular prevention in women: A narrative review from the Italian Society of Cardiology working groups on “Cardiovascular Prevention, Hypertension and peripheral circulation” and on “Women Disease.” J Cardiovasc Med. 2019 Sep;20(9):575–83.
7. Rosano GMC, Vitale C, Marazzi G, Volterrani M. Menopause and cardiovascular disease: the evidence. Climacteric. 2007 Jan;10(sup1):19–24.
8. Perez CC. Invisible Women: Exposing Data Bias in a World Designed for Men. Random House; 2019.
9. Costello JT, Bieuzen F, Bleakley CM. Where are all the female participants in Sports and Exercise Medicine research? Eur J Sport Sci. 2014 Nov 17;14(8):847–51.
10. Vitale C, Fini M, Spoletini I, Lainscak M, Seferovic P, Rosano GM. Under-representation of elderly and women in clinical trials. Int J Cardiol. 2017 Apr;232:216–21.
11. Börjesson M, Onerup A, Lundqvist S, Dahlöf B, Borjesson M, Onerup A, et al. Physical activity and exercise lower blood pressure in individuals with hypertension: Narrative review of 27 RCTs. Br J Sports Med. 2016 Mar;50(6):356–61.
12. Brook RD, Appel LJ, Rubenfire M, Ogedegbe G, Bisognano JD, Elliott WJ, et al. Beyond Medications and Diet: Alternative Approaches to Lowering Blood Pressure. Hypertension. 2013 Jun;61(6):1360–83.
13. Arnett DK, Blumenthal RS, Albert MA, Buroker AB, Goldberger ZD, Hahn EJ, et al. 2019 ACC/AHA Guideline on the Primary Prevention of Cardiovascular Disease: A Report of the American College of Cardiology/American Heart Association Task Force on Clinical Practice Guidelines. Circulation. 2019 Sep;140(11):e596–646.
14. Pedersen BK, Saltin B. Exercise as medicine - Evidence for prescribing exercise as therapy in different chronic diseases. Scand J Med Sci Sport. 2015 Dec;25:1–72.
15. Lee D, Artero EG, Sui X, Blair SN. Mortality trends in the general population: the importance of cardiorespiratory fitness. J Psychopharmacol. 2010 Nov;24(4 Suppl):27–35.
16. Liu Y, Lee DC, Li Y, Zhu W, Zhang R, Sui X, et al. Associations of Resistance Exercise with Cardiovascular Disease Morbidity and Mortality. Med Sci Sports Exerc. 2019 Mar;51(3):499–508.
17. El-Kotob R, Ponzano M, Chaput JP, Janssen I, Kho ME, Poitras VJ, et al. Resistance training and health in adults: an overview of systematic reviews. Appl Physiol Nutr Metab. 2020 Oct;45(10 (Suppl. 2)):S165–79.
18. Pollock ML, Franklin BA, Balady GJ, Chaitman BL, Fleg JL, Fletcher B, et al. Resistance exercise in individuals with and without cardiovascular disease: Benefits, rationale, safety, and prescription: An advisory from the Committee on Exercise, Rehabilitation, and Prevention, Council on Clinical Cardiology, American Heart Associati. Circulation. 2000 Feb;101(7):828–33.
19. Visseren FLJ, Mach F, Smulders YM, Carballo D, Koskinas KC, Bäck M, et al. 2021 ESC Guidelines on cardiovascular disease prevention in clinical practice. Eur Heart J. 2021 Sep 7;42(34):3227–337.
20. MacDonald H V., Johnson BT, Huedo-Medina TB, Livingston J, Forsyth KC, Kraemer WJ, et al. Dynamic resistance training as stand-alone antihypertensive lifestyle therapy: A meta-analysis. J Am Heart Assoc. 2016 Oct;5(10).
21. Cornelissen VA, Smart NA. Exercise training for blood pressure: a systematic review and meta-analysis. J Am Heart Assoc. 2013 Feb;2(1):e004473.
22. Cornelissen VA, Fagard RH, Coeckelberghs E, Vanhees L. Impact of resistance training on blood pressure and other cardiovascular risk factors: A meta-analysis of randomized, controlled trials. Hypertension. 2011 Nov;58(5):950–8.
23. Cornelissen VA, Fagard RH. Effect of resistance training on resting blood a pressure: A meta-analysis of randomized controlled trials. J Hypertens. 2005;23(2):251–9.
24. Kelley GA, Kelley KS. Progressive resistance exercise and resting blood pressure: A meta- analysis of randomized controlled trials. Hypertension. 2000;35(3):838–43.
25. Pescatello LS, Franklin BA, Fagard R, Farquhar WB, Kelley GA, Ray CA. Exercise and Hypertension. Med Sci Sport Exerc. 2004 Mar;36(3):533–53.
26. De Sá CA, Catani D, Cardoso AM, Da Silva Grigoletto ME, Battiston FG, Corralo VS. Resistance training affects the hemodynamic parameters of hypertensive and normotensive women differently, and regardless of performance improvement. J Exerc Sci Fit. 2020 Sep;18(3):122.
27. Cornelissen VA, Fagard RH, Coeckelberghs E, Vanhees L. Impact of Resistance Training on Blood Pressure and Other Cardiovascular Risk Factors. Hypertension. 2011 Nov;58(5):950–8.
28. Horsman HM, Peebles KC, Galletly DC, Tzeng YC. Cardiac baroreflex gain is frequency dependent: Insights from repeated sit-to-stand maneuvers and the modified Oxford method. Appl Physiol Nutr Metab. 2013 Jun;38(7):753–9.
29. Braith RW, Stewart KJ. Resistance exercise training: Its role in the prevention of cardiovascular disease. Vol. 113, Circulation. Lippincott Williams & Wilkins; 2006. p. 2642–50.
30. McCartney N, McKelvie RS, Martin J, Sale DG, MacDougall JD. Weight-training-induced attenuation of the circulatory response of older males to weight lifting. J Appl Physiol. 1993;74(3):1056–60.
31. Fecchio RY, Brito LC, Peçanha T, de Moraes Forjaz CL. Potential Mechanisms Behind the Blood Pressure–Lowering Effect of Dynamic Resistance Training. Vol. 23, Current Hypertension Reports. Curr Hypertens Rep; 2021.
32. Fagard RH, Cornelissen VA. Effect of exercise on blood pressure control in hypertensive patients. Eur J Cardiovasc Prev Rehabil. 2007 Feb;14(1):12–7.
33. Loaiza-Betancur AF, Chulvi-Medrano I, Díaz-López VA, Gómez-Tomás C. The effect of exercise training on blood pressure in menopause and postmenopausal women: A systematic review of randomized controlled trials. Vol. 149, Maturitas. Elsevier; 2021. p. 40–55.
34. Kenney MJ, Seals DR. Postexercise hypotension: Key features, mechanisms, and clinical significance. Hypertension. 1993 Nov;22(5):653–64.
35. Marçal IR, Goessler KF, Buys R, Casonatto J, Ciolac EG, Cornelissen VA. Post-exercise Hypotension Following a Single Bout of High Intensity Interval Exercise vs. a Single Bout of Moderate Intensity Continuous Exercise in Adults With or Without Hypertension: A Systematic Review and Meta-Analysis of Randomized Clinical Trials. Front Physiol. 2021 Jun;12.
36. Brito LC, Queiroz ACC, Forjaz CLM. Influence of population and exercise protocol characteristics on hemodynamic determinants of post-aerobic exercise hypotension. Brazilian J Med Biol Res [Internet]. 2014 Aug;47(8):626–36.
37. Teixeira L, Ritti-Dias RM, Tinucci T, Mion Jr. D, De Moraes Forjaz CL, Mion Júnior D, et al. Post-concurrent exercise hemodynamics and cardiac autonomic modulation. Eur J Appl Physiol. 2011 Sep;111(9):2069–78.
38. Casonatto J, Goessler KF, Cornelissen VA, Cardoso JR, Polito MD. The blood pressure-lowering effect of a single bout of resistance exercise: A systematic review and meta-analysis of randomised controlled trials. Eur J Prev Cardiol. 2016 Nov;23(16):1700–14.
39. Rúa-Alonso M, Mayo X, Mota J, Kingsley JD, Iglesias-Soler E. A short set configuration attenuates the cardiac parasympathetic withdrawal after a whole-body resistance training session. Eur J Appl Physiol. 2020;120(8):1905–19.
40. Río-Rodríguez D, Iglesias-Soler E, Fernández Del Olmo M. Set Configuration in Resistance Exercise: Muscle Fatigue and Cardiovascular Effects. PLoS One. 2016;11(3):e0151163.
41. Mayo X, Iglesias-Soler E, Carballeira-Fernández E, Fernández-Del-Olmo M. A shorter set reduces the loss of cardiac autonomic and baroreflex control after resistance exercise. Eur J Sport Sci. 2015;16(8):996–1004.
42. Iglesias-Soler E, Boullosa DA, Carballeira E, Sánchez-Otero T, Mayo X, Castro-Gacio X, et al. Effect of set configuration on hemodynamics and cardiac autonomic modulation after high-intensity squat exercise. Clin Physiol Funct Imaging. 2015 Jul;35(4):250–7.
43. Mayo X, Iglesias-Soler E, Fariñas-Rodríguez J, Fernández-Del-Olmo M, Kingsley JD. Exercise Type Affects Cardiac Vagal Autonomic Recovery After a Resistance Training Session. J strength Cond Res. 2016 Sep;30(9):2565–73.
44. Mayo X, Iglesias-Soler E, Kingsley JD, Dopico X. Interrepetition Rest Set Lacks the V-Shape Systolic Pressure Response Advantage during Resistance Exercise. Sports. 2017 Dec;5(4):90.
45. Paz GA, Iglesias-Soler E, Willardson JM, Maia M de F, Miranda H. Postexercise Hypotension and Heart Rate Variability Responses Subsequent to Traditional, Paired Set, and Superset Resistance Training Methods. J strength Cond Res. 2019 Sep;33(9):2433–42.
46. Mayo X, Iglesias-Soler E, Fustes-Piñeiro S, González-Hernández R. The effect of set configuration and type of resistance exercise on recovery blood pressure. In: 4th International Conference on Human Performance Development through Strength and Conditioning, NSCA 2014. Murcia, Spain; 2014.
47. MacDougall JD, Tuxen D, Sale DG, Moroz JR, Sutton JR. Arterial blood pressure response to heavy resistance exercise. J Appl Physiol. 1985 Mar;58(3):785–90.
48. Mccartney N. Acute responses to resistance training and safety. Med Sci Sport Exerc. 1999 Jan;31(1):31–7.
49. Rosenwinkel ET, Bloomfield DM, Arwady MA, Goldsmith RL. Exercise and autonomic function in health and cardiovascular disease. Cardiol Clin. 2001;19(3):369–87.
50. Bjarnason-Wehrens B, Mayer-Berger W, Meister ER, Baum K, Hambrecht R, Gielen S. Recommendations for resistance exercise in cardiac rehabilitation. Recommendations of the German Federation for Cardiovascular Prevention and Rehabilitation. Eur J Prev Cardiol. 2004;11(4):352–61.
51. Gomides R, Dias R, Souza D, Costa L, Ortega K, Mion D, et al. Finger blood pressure during leg resistance exercise. Int J Sports Med. 2010 Aug;31(08):590–5.
52. de Sousa NM, Magosso RF, Dipp T, Plentz RD, Marson RA, Montagnolli AN, et al. Continuous blood pressure response at different intensities in leg press exercise. Eur J Prev Cardiol. 2014 Nov;21(11):1324–31.
53. Sale DG, Moroz DE, McKelvie RS, MacDougall JD, McCartney N. Effect of Training on the Blood Pressure Response to Weight Lifting. Can J Appl Physiol. 1994 Mar;19(1):60–74.
54. Falkel JE, Fleck SJ, Murray TF. Comparison of Central Hemodynamics Between Powerlifters and Bodybuilders During Resistance Exercise. J Strength Cond Res. 1992;6(1):24.
55. Nery S de S, Gomides RS, da Silva GV, Forjaz CL de M, Mion D, Tinucci T, et al. Intra-arterial blood pressure response in hypertensive subjects during low- and high-intensity resistance exercise. Clinics. 2010;65(3):271–7.
56. Lamotte M, Strulens G, Niset G, Van De Borne P. Influence of different resistive training modalities on blood pressure and heart rate responses of healthy subjects. Isokinet Exerc Sci. 2005 Jan;13(4):273–7.
57. Lamotte M, Fournier F, Vanissum A, Van De Borne P. Influence of rest period duration between successive muscular strength sets on acute modifications of blood pressure and heart rate in the healthy subject. Isokinet Exerc Sci. 2006;14(4):349–55.
58. Lamotte M, Niset G, van de Borne P. The effect of different intensity modalities of resistance training on beat-to-beat blood pressure in cardiac patients. Eur J Cardiovasc Prev Rehabil. 2005 Feb;12(1):12–7.
59. Lamotte M, Fleury F, Pirard M, Jamon A, Borne P van de. Acute cardiovascular response to resistance training during cardiac rehabilitation: effect of repetition speed and rest periods. Eur J Cardiovasc Prev Rehabil. 2010 Jun;17(3):329–36.
60. Williams MA, Haskell WL, Ades PA, Amsterdam EA, Bittner V, Franklin BA, et al. Resistance Exercise in Individuals With and Without Cardiovascular Disease: 2007 Update. Circulation. 2007 Jul 31;116(5):572–84.
61. Tufano JJ, Brown LE, Haff GG. Theoretical and Practical Aspects of Different Cluster Set Structures: A Systematic Review. Vol. 31, Journal of Strength and Conditioning Research. NSCA National Strength and Conditioning Association; 2017. p. 848–67.
62. Mayo X, Iglesias-Soler E, Fernández-Del-Olmo M. Effects of Set Configuration of Resistance Exercise on Perceived Exertion. Percept Mot Skills. 2014 Dec;119(3):825–37.
63. Mayo X, Iglesias-Soler E, Kingsley JD. Perceived Exertion Is Affected by the Submaximal Set Configuration Used in Resistance Exercise. J strength Cond Res. 2019 Feb;33(2):426–32.
64. Iglesias-Soler E, Boullosa DA, Dopico X, Carballeira E. Analysis of Factors That Influence the Maximum Number of Repetitions in Two Upper-Body Resistance Exercises: Curl Biceps and Bench Press. J Strength Cond Res. 2010 Jun;24(6):1566–72.
65. Iglesias-Soler E, Carballeira E, Sánchez-Otero T, Mayo X, Jiménez A, Chapman ML, et al. Acute effects of distribution of rest between repetitions. Int J Sports Med. 2012 May;33(05):351–8.
66. Fariñas J, Mayo X, Giraldez-García MA, Carballeira E, Fernandez-Del-Olmo M, Rial-Vázquez J, et al. Set Configuration in Strength Training Programs Modulates the Cross Education Phenomenon. J Strength Cond Res. 2019;(12):1.
67. Iglesias-Soler E, Carballeira E, Sanchez-Otero T, Mayo X, Jimenez a, Chapman ML. Acute Effects of Distribution of Rest between Repetitions. Int J Sports Med. 2012;33:351–8.
68. Iglesias-Soler E, Carballeira E, Sánchez-Otero T, Mayo X, Fernández-del-Olmo M. Performance of maximum number of repetitions with cluster-set configuration. Int J Sports Physiol Perform. 2014 Jul;9(4):637–42.
69. Rial-Vázquez J, Mayo X, Tufano JJJ, Fariñas J, Rúa-Alonso M, Iglesias-Soler E. Cluster vs. traditional training programmes: changes in the force–velocity relationship. Sport Biomech. 2020 Mar;5:1–19.
70. Baum K, Ruther T, Essfeld D. Reduction of Blood Pressure Response During Strength Training Through Intermittent Muscle Relaxations.pdf. Int J Sport Med. 2003;24(6):441–5.
71. Veloso Ú, Monteiro W, Farinatti P. Do continuous and intermittent exercises sets induce similar cardiovascular responses in the elderly women? Rev Bras Med do Esporte. 2003;9(2):85–90.
72. Coelho CW, Hamar D, de Araújo CGS. Physiological responses using 2 high-speed resistance training protocols. J strength Cond Res. 2003 May;17(2):334–7.
73. Ribeiro-Torres O, de Sousa AFM, Iglesias-Soler E, Fontes-Villalba M, Zouhal H, Carré F, et al. Lower Cardiovascular Stress during Resistance Training Performed with Inter-Repetition Rests in Elderly Coronary Patients. Medicina (B Aires). 2020 May;56(6):264.
74. Malik M. Heart rate variability: standards of measurement, physiological interpretation and clinical use. Task Force of the European Society of Cardiology and the North American Society of Pacing and Electrophysiology. Circulation. 1996 Mar;93(5):1043–65.
75. Parati G, Saul JP, Rienzo M Di, Mancia G. Spectral Analysis of Blood Pressure and Heart Rate Variability in Evaluating Cardiovascular Regulation. Hypertension. 1995;25(6):1276–86.
76. Stuckey MI, Tordi N, Mourot L, Gurr LJ, Rakobowchuk M, Millar PJ, et al. Autonomic recovery following sprint interval exercise. Scand J Med Sci Sports. 2012 Dec;22(6):756–63.
77. Marasingha-Arachchige SU, Rubio-Arias JÁ, Alcaraz PE, Chung LH. Factors that affect heart rate variability following acute resistance exercise: A systematic review and meta-analysis. J Sport Heal Sci. 2020 Nov;
78. Kingsley JD, Figueroa A. Acute and training effects of resistance exercise on heart rate variability. Clin Physiol Funct Imaging. 2014 May;36(3):179–87.
79. Kingsley JD, Tai YL, Marshall EM, Glasgow A, Oliveira R, Parks JC, et al. Autonomic modulation and baroreflex sensitivity after acute resistance exercise: responses between sexes. J Sports Med Phys Fitness. 2019;59(6):1036–44.
80. Machado MV, Barbosa T de PC, Chrispino TC, Junqueira das Neves F, Rodrigues GD, Soares PP da S, et al. Cardiovascular and Autonomic Responses after a Single Bout of Resistance Exercise in Men with Untreated Stage 2 Hypertension. Int J Hypertens. 2021 Mar;2021:6687948.
81. Heffernan KS, Collier SR, Kelly EE, Jae SY, Fernhall B. Arterial stiffness and baroreflex sensitivity following bouts of aerobic and resistance exercise. Int J Sports Med. 2007;28(3):197–203.
82. Mota MRMR, Pardono E, Lima LCJ, Arsa G, Bottaro M, Campbell CSG, et al. Effects of treadmill running and resistance exercises on lowering blood pressure during the daily work of hypertensive subjects. J strength Cond Res. 2009 Nov;23(8):2331–8.
83. Niemelä THT, Kiviniemi AMAAM, Hautala AJA, Salmi JAJ, Linnamo V, Tulppo MMP. Recovery pattern of baroreflex sensitivity after exercise. Med Sci Sports Exerc. 2008 May;40(5):864–70.
84. Albert CM, Mittleman MA, Chae CU, Lee IM, Hennekens CH, Manson JE. Triggering of sudden death from cardiac causes by vigorous exertion. N Engl J Med. 2000 Nov;343(19):1355–61.
85. Mittleman MA, Maclure M, Tofler GH, Sherwood JB, Goldberg RJ, Muller JE. Triggering of Acute Myocardial Infarction by Heavy Physical Exertion - Protection against Triggering by Regular Exertion. N Engl J Med. 1993 Dec;329(23):1677–83.
86. Fred HL. More on weightlifting injuries. Texas Hear Inst J. 2014 Aug;41(4):453–4.
87. Franklin BA, Thompson CPD, Al-Zaiti SS, Albert CM, Hivert M-FF, Levine BD, et al. Exercise-Related Acute Cardiovascular Events and Potential Deleterious Adaptations Following Long-Term Exercise Training: Placing the Risks Into Perspective–An Update: A Scientific Statement From the American Heart Association. Circulation. 2020 Mar;141(13):E705–36.
