# Supplementary material for: Acute cardiovascular responses of postmenopausal women to resistance training sessions differing in set configuration: A study protocol for a crossover trial
Source: PLoS One. 2024 Oct 14;19(10):e0311524. doi: 10.1371/journal.pone.0311524 (PMC11472946; doi:10.1371/journal.pone.0311524)
Supplement: Attachment — Submitted filename: REBUTTAL LETTER.docx [file pone.0311524.s005.docx]

**REBUTTAL LETTER**

1. **Please ensure that your manuscript meets PLOS ONE's style requirements, including those for file naming. The PLOS ONE style templates can be found at**

RESPONSE: We have meticulously adhered to the template and ensured that all files are named in accordance with the correct format as outlined in the style guidelines.

**2. Thank you for stating the following financial disclosure:**

***"This work is part of the project PID2021-124277OB-I00 funded by MCIN/AEI/10.13039/501100011033/FEDER, UE. JR-V and MR-A acknowledge the financial support received from the Spanish Ministry of Universities through the Grants for the Requalification of the Spanish University System under the Postdoctoral Margarita Salas Program (JR-V: RSU.UDC.MS10; and MR-A: RSUC.UDC.MS09), funded by the European Union – Next Generation."***

***Please state what role the funders took in the study. If the funders had no role, please state: ""The funders had no role in study design, data collection and analysis, decision to publish, or preparation of the manuscript.""***

***If this statement is not correct you must amend it as needed.***

***Please include this amended Role of Funder statement in your cover letter; we will change the online submission form on your behalf.***

RESPONSE: The statement you suggested has been incorporated into the revised version of the cover letter.

**3. Your ethics statement should only appear in the Methods section of your manuscript. If your ethics statement is written in any section besides the Methods, please delete it from any other section.**

RESPONSE: The Ethical Statement has been removed from other sections and now appears exclusively in the Methods section.

**4. We note that Figure 2 in your submission contain copyrighted images. All PLOS content is published under the Creative Commons Attribution License (CC BY 4.0), which means that the manuscript, images, and Supporting Information files will be freely available online, and any third party is permitted to access, download, copy, distribute, and use these materials in any way, even commercially, with proper attribution.**

RESPONSE: We have removed the potentially copyrighted images and provided a new figure with images that we created ourselves and are free of copyright.

**5. We are unable to open your Supporting Information file SUPPORTING INFORMATION.rar. Please kindly revise as necessary and re-upload.**

RESPONSE: We regret this issue. We have now uploaded the Supporting Information as individual files to avoid any problems with the compressed folder.

**6. Please review your reference list to ensure that it is complete and correct. If you have cited papers that have been retracted, please include the rationale for doing so in the manuscript text, or remove these references and replace them with relevant current references. Any changes to the reference list should be mentioned in the rebuttal letter that accompanies your revised manuscript. If you need to cite a retracted article, indicate the article’s retracted status in the References list and also include a citation and full reference for the retraction notice.**

RESPONSE: We have reviewed the reference list and confirmed that all cited papers are accurate, and none have been retracted.

**Reviewers' comments:**

***Reviewer #1: Very nice. Considering this is a protocol paper, I would say everything is well-described and the rationale is there. To me, those are the most important aspects for such a paper. Clearly, the "discussion" pointes are theoretical, but should be well-defined and connected to the current literature when the scientific publications come out. I look forward to the paper(s) in the future. Keep up the good work.***

RESPONSE: We sincerely appreciate the reviewer’s comments. Thank you very much for your valuable feedback

***Reviewer #2: In this study protocol, a 4 segment randomized crossover design is being proposed to evaluate the impact on resistance training set configuration on arterial stiffness, hemodynamic variables, cardiac autonomic, modulation, baroreflex sensitivity, sympathetic vasomotor tone, resting oxygen uptake, perceived effort, mechanical performance, heart rate, and lactatemia, along with comparisons between the normotensive and hypertensive groups.***

RESPONSE: We thank the reviewer for their comments and suggestions. Please find below our responses to your specific comments.

**Minor revisions:**

1- **Line 180: State the statistical testing method which achieves 80% power.**

RESPONSE: Thank you for your comment. We conducted two types of power calculations for ANOVA tests: one for the interaction in a 4 x 2 repeated-measures ANOVA (set configuration * time) and another for the interaction in a 4 x 2 ANOVA involving a within-subject factor (i.e., set configuration 1-3 and control session), and a between-subjects factor (i.e., hypertensive and normotensive). For the same conditions, the former analysis required 49 participants, while the latter needed a total sample of 50 (25 per group). Therefore, we decided to report the more demanding test. Additionally, as suggested by the reviewer, we have specified in the revised manuscript the test for which the sample size was calculated. *“A priori power analysis was conducted using G * Power V3 (Universität Kiel, Düsseldorf, Germany). Considering the research design, the sample size calculation was carried out for a statistical power of 80% (1– β) and assumed Type Error I of 0.05 in order to detect a small effect size (f = 0.12) for the interaction between the within-subject factor (i.e., set configuration 1-3 and control session) and between-subjects factor (i.e., normotensive and hypertensive groups) in an Analysis of Variance test (ANOVA), and assuming a correlation of 0.75 between repeated measures”*

2- **Identify the software that will be used to capture the data.**

RESPONSE: The software to be used for capturing each variable has been added as requested.
